# Three Phases of CD8 T Cell Response in the Lung Following H1N1 Influenza Infection and Sphingosine 1 Phosphate Agonist Therapy

**DOI:** 10.1371/journal.pone.0058033

**Published:** 2013-03-22

**Authors:** Melanie P. Matheu, John R. Teijaro, Kevin B. Walsh, Milton L. Greenberg, David Marsolais, Ian Parker, Hugh Rosen, Michael B A. Oldstone, Michael D. Cahalan

**Affiliations:** 1 Department of Physiology and Biophysics and the Center for Immunology, University of California Irvine, Irvine, California, United States of America; 2 Department of Immunology and Microbial Science, The Scripps Research Institute, La Jolla, California, United States of America; 3 Department of Chemical Physiology, The Scripps Research Institute, La Jolla, California, United States of America; 4 Department of Medicine, Faculty of Medicine, Laval University; IUCPQ Research Center, Québec, Québec, Canada; 5 Department of Neurobiology and Behavior, University of California Irvine, Irvine, California, United States of America; La Jolla Institute for Allergy and Immunology, United States of America

## Abstract

Influenza-induced lung edema and inflammation are exacerbated by a positive feedback loop of cytokine and chemokine production termed a ‘cytokine storm’, a hallmark of increased influenza-related morbidity and mortality. Upon infection, an immune response is rapidly initiated in the lungs and draining lymph node, leading to expansion of virus-specific effector cells. Using two-photon microscopy, we imaged the dynamics of dendritic cells (DC) and virus-specific eGFP^+^CD8^+^ T cells in the lungs and draining mediastinal lymph nodes during the first two weeks following influenza infection. Three distinct phases of T cell and CD11c^+^ DC behavior were revealed: 1) Priming, facilitated by the arrival of lung DCs in the lymph node and characterized by antigen recognition and expansion of antigen-specific CD8^+^ T cells; asymmetric T cell division in contact with DCs was frequently observed. 2) Clearance, during which DCs re-populate the lung and T cells leave the draining lymph node and re-enter the lung tissue where enlarged, motile T cells come into contact with DCs and form long-lived interactions. 3) Maintenance, characterized by T-cell scanning of the lung tissue and dissociation from local antigen presenting cells; the T cells spend less time in association with DCs and migrate rapidly on collagen. A single dose of a sphingosine-1-phosphate receptor agonist, AAL-R, sufficient to suppress influenza-induced cytokine-storm, altered T cell and DC behavior during influenza clearance, delaying T cell division, cellular infiltration in the lung, and suppressing T-DC interactions in the lung. Our results provide a detailed description of T cell and DC choreography and dynamics in the lymph node and the lung during influenza infection. In addition, we suggest that phase lags in T cell and DC dynamics induced by targeting S1P receptors in vivo may attenuate the intensity of the immune response and can be manipulated for therapeutic benefit.

## Introduction

Influenza A infection can be lethal, because of massive inflammation which results in lung-tissue damage from extensive epithelial autophagy and intra-alveolar edema that lead to acute respiratory distress syndrome (ARDS) [1], [2], [3]. During pathological H1N1 influenza A infection, an aggressive immune response is initiated by lung endothelial cell cytokines [1], [2], which recruit innate and effector T cells that together contribute to epithelial cell death and a dysregulated positive feedback loop of cytokine production termed a ‘cytokine storm’ [3], [4]. In humans, this rapid and highly reactive immune response is the underlying cause of respiratory complications that persist long after viral clearance. Patients hospitalized and diagnosed with H1N1-induced ARDS frequently suffer significant reduction in lung function and health-related quality of life for up to twelve months [5], [6]. In addition to palliative therapy, a variety of new pharmaceutical targets designed to quell induction of the ‘cytokine storm’ are being investigated as potential therapies in patients suffering from influenza A infection. In animal models, suppression of early cytokine induction by targeting sphingosine-1-phosphate (S1P) receptors using agonists AAL-R (S1P_1,3–5_) or CYM-5442 (S1P_1_) has been demonstrated to hinder development of a cytokine storm, early innate cell infiltration, and effector CD8^+^ T cell responses while significantly increasing animal survival rates without altering the kinetics of viral clearance [1], [7], [8]. In contrast, corticosteroids currently used to treat influenza-induced ARDS broadly suppress immune responses and may ultimately be detrimental [9], [10].

Influenza infection in the lung and upper airways initiates an adaptive immune response to peripheral infection by inducing long-range migration of antigen-bearing, tissue-resident dendritic cells (DCs) to the draining (mediastinal) lymph node [11]. Two-photon imaging of T cell-APC interactions in living tissues has previously yielded tremendous insights into T cell behavior during activation in the context of antigen-specific immune responses that produce protective immunity [12], [13]. Here, we applied 2-photon imaging to elucidate the dynamics of CD11c^+^ dendritic cell and antigen-specific naïve CD8^+^ T behavior in the lung and mediastinal lymph node in response to mouse-adapted H1N1 influenza A virus (A/WSN/33). Our model of influenza infection allows us to image and analyze the progression of an immune response to influenza A infection through the classically described phases of priming, clonal expansion and contraction. Here, we add to this classical understanding of the adaptive immune response by describing these three phases of an immune response in terms of CD8^+^ T cell and DC behavior and interactions. We further investigated the effects of S1P agonist treatment on cellular dynamics in the lung and draining lymph node and discovered phase lags in the cellular behavior that may lead to therapeutic benefit.

## Results

### Imaging DCs in the lung

**Figure 1:**
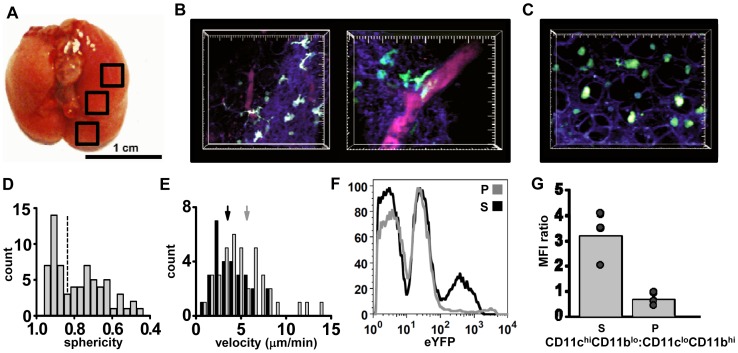
Imaging lung tissue in CD11c-eYFP^+^ mice. (A) Whole mouse lung inflated with 10% low-melt agarose, trachea folded forward, and general regions of imaging marked by black squares. (B) Two views of the lung from a CD11c-eYFP^+^ animal taken in the lower left lobe, 0–50 μm below the dense collagen network at the lung surface. The surface of the lung is identifiable by second harmonic generation (violet-blue) of the dense collagen network (20–30 µm thick) before giving way to alveolar spaces (parenchyma). Blood vessels were highlighted by injection of 655 nm Q-dots (i.v.) 5 min prior to lung harvest (right; large tick marks  = 10 µm). (C) Rounded eYFP-dim cells 150 µm below the surface in the parenchyma of the lung. Large tick marks in B and C = 10 µm. (D) Sphericity of eYFP^+^ cells imaged in all locations of the lungs; note the bimodal distribution (1 =  perfect sphere). (E) Distributions of mean cell velocities in eYFP-dim cells of low (grey bars) and high (black bars) sphericity (>0.85 and <0.85; as indicated by dashed line in D). Arrows indicate mean velocities of low and high sphericity cells (5.7±0.45 and 3.5±0.34 μm/min, respectively; p<0.01). (F) FACS analysis after micro-dissection of agarose-inflated lung tissue to separate the imaged surface (S) of the lung from the parenchyma (P). (G) The ratio of CD11c^hi^CD11b^lo^ vs CD11c^lo^CDl11b^hi^ cells is significantly higher near the lung surface than in the parenchyma (mean  = 3.2±0.6 and 0.7±0.3, respectively, p = 0.03). Each dot represents an individual cell, bar represents the mean of 3 trials. Representative images taken from 3 separate experiments.

To image the immune response to influenza infection over the course of two weeks, we developed a novel, semi-intact lung preparation that allowed for consistent access to the all regions of the lung, while maintaining cell viability and structural integrity. To achieve this, the mouse trachea was intubated immediately after sacrifice and the lungs were slowly filled with low-melt agarose, slices distal from the imaging site were made and imaging was performed in the areas outlined by black boxes (Figure 1A). Agarose maintained structural integrity by keeping the lungs inflated while allowing for deep-tissue imaging by eliminating air pockets that differ in refractive index from in situ imaging media. After inflation of the lungs with low melt agarose, whole lung tissue was removed intact and imaged. CD11c-eYFP mice [14] were used in all imaging experiments. We observed eYFP-bright cells near the surface of the lung (20–50 μm deep), and on occasion near small blood vessels. These cells exhibited characteristic properties of DCs, demonstrating an extended cell morphology and active sampling of the local environment; (Figure 1B, Video S1, left). In contrast, a different population of eYFP-dim cells was found deeper in the lung tissue (50–200 μm below the surface) and sometimes within alveolar spaces. These eYFP-dim cells had a spherical morphology consistent with alveolar macrophages (Figure 1C, Video S1, right). These findings are consistent with previously described DC and alveolar macrophage morphology in live lung imaging using a suction window preparation [15]. The eYFP^+^ cells had a bi-modal distribution of sphericity (where 1  =  perfect sphere; Figure 1D). The two populations of cells showed significantly different velocities: macrophage-like cells (sphericity >0.85) had higher average velocity than dendritic-like cells (sphericity <0.85; Figure 1E). The more spherical cells found in the deeper regions of the lung tended to be smaller in volume (1250±232 μm^3^) than less spherical cells (4093±232 μm^3^, p<0.001). Therefore, the phenotype of the relatively smaller, more spherical eYFP-dim cells is concordant with their being alveolar macrophages, whereas the morphology of eYFP-bright cells found at the surface of the lung and near blood vessels is consistent with previous reports describing CD11c^+^ lung DCs [15].

To determine the phenotype of lung-resident populations of eYFP^+^ cells we analyzed expression of common DC markers. In order to examine cells near the surface of the lung, 50–200 μm sections of tissue from the lung surface of CD11c-eYFP mice were separated by micro-dissection and homogenized for whole-tissue FACS. The outer lung contained both eYFP^hi^ and eYFP^lo^ cells, whereas internal lung tissue contained only eYFP^lo^ cells (Figure 1F). Furthermore, the ratio of CD11c^hi^ CD11b^lo^ to CD11c^lo^ CD11b^hi^ cells was nearly three times higher at the surface of the lung relative to the parenchyma (Figure 1G). Finally, because influenza clearance is dependent upon a migratory subtype of dendritic cell that is CD11c^hi^CD11b^lo^CD103^+^ [11], [16], we examined the distribution of CD11c^hi^CD103^+^ dendritic cells. We found that the CD103^+^ population was enriched at the surface lung tissue and CD103 expression correlated with high expression of CD11c and eYFP (Figure S1A, B). These results indicate that the actively probing, motile eYFP^hi^ cells seen at the lung surface are migratory DCs that are essential for priming CD8^+^ T cell responses in the draining lymph node [11], [16], [17].

### Phase 1 (Days 1–3): arrival of highly motile DCs in the draining lymph node and T cell priming

**Figure 2:**
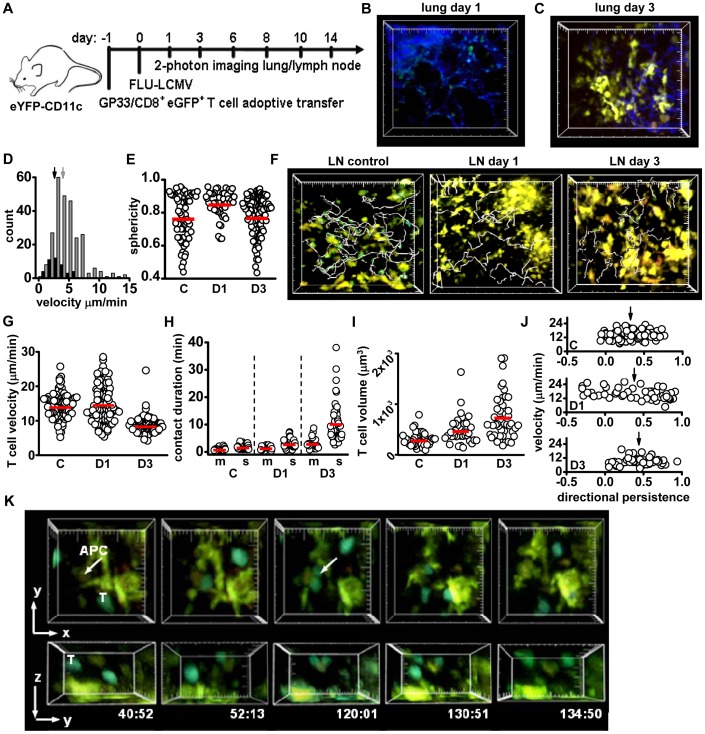
Phase 1: Events in the lung and lymph node during the Priming phase. (A) Time-line of infection and tissue harvesting for influenza imaging. B) Lung tissue surface image 24 h after infection showing clearance of the eYFP-bright cells (cf. Fig. 1B); small eYFP-dim cells remain. Large tick marks in B, C, F  = 20 μm. (C) Three days after infection, eYFP-bright cells form clusters just below the collagen-rich surface. (D) The velocity of eYFP^+^ DCs in the lung on days 1 (black) and 3 (grey). Mean velocity is significantly higher on day 3 (5.1±0.1 vs. 2.6±0.2 μm/min, respectively; p<0.01). (E) Sphericity of the eYFP^+^ cells before infection (C, 0.77±0.02, p<0.01), remaining in the lung on day 1 (D1, 0.85±0.01), and on day 3 after infection (D3, 0.78±0.01, p<0.01). Circles are measurements from individual cells, red bars  =  mean. (F) Tracks (grey) of eGFP^+^ CD8^+^ T cells (green) in mediastinal lymph nodes with no infection (control), and on days 1 and 3 post-infection; eYFP-CD11c^+^ DCs (yellow) are also seen. Tracks represent 20 min of T cell movement for control and day 3, and 59 min of T cell movement on day 1; track times differed to allow for visualization of rare T cells on day 1. (G) Mean lymph node T cell velocities in control, day 1, and day 3 post-infection (respectively, 14.3±0.3, 14.9±0.5, and 8.6±0.2 μm/min). Control and day 1 T cell values are not statistically different (p = 0.9); both have higher velocity than T cells on day 3 (p<0.01 for both). (H) T cell-DC contact durations with motile (m) or sessile (s) DCs. Compared to control, contact durations increase significantly for motile DCs on day 3 (3.0±0.4 min, p<0.01 for both motile DC-T comparisons), and for sessile DCs on day 1 (3.0+0.2 min, p<0.01) and day 3 (10.3±1.0 min, p<0.01 for both sessile DC-T comparisons). (I) The volume of T cells in the lymph node for control T cells (335±24 μm^3^), on day 1 (485±43 μm^3^) and day 3 (1040±138 μm^3^). Note 1 μm^3^  = 1 femtoliter. p<0.01 for all comparisons. (J) Average track directional persistence (dp) plotted against velocity for control (mean track dp  = 0.33±0.02), day 1 (dp  = 0.38±0.05), and day 3 (dp  = 0.44±0.03, p<0.01 both comparisons) T cells. On day 1, T cells have a negative correlation with velocity (Pearson's correlation coefficient PCC of −0.4, p = 0.004), and correlation between velocity and directional persistence is not evident in control and day 3 analysis (PCC  = 0.1 and 0.13 respectively, p>0.2 in both samples). (K) Example of T cell division on day 3 while in contact with a sessile APC (large tick marks  = 10 μm). Data from each tissue and day were pooled from 4–6 separate experiments; each individual dot represents the behavior of a single cell, bars represent the mean.

To image T cell priming and effector responses to influenza in the lung, mouse-adapted H1N1 influenza virus expressing the LCMV protein GP33 (A/WSN/33; FLU-LCM) was used to infect CD11c-eYFP mice 24 h after the adoptive transfer of 5×10^4^ naïve GP33-specific eGFP^+^CD8^+^ (P14) T cells [18] (Figure 2A). Mediastinal lymph nodes and lungs were imaged on days 1 through 14 after infection at the indicated times. The kinetics of viral clearance replicated time courses previously reported in studies of human and murine immune responses to FLU-LCMV, in which complete clearance was achieved by day 8 post-infection [7], [19], [20]. Influenza infection induced the migration of DCs from the lung, corresponding to the arrival of highly motile DCs in the draining lymph node. Within 24 h, no eYFP-bright DCs were apparent near the surface of the lung (Figure 2B, Video S2). On day 3 post-infection, the number of visible DC-like, eYFP-bright cells in the lung increased in most animals imaged (Figure 2C, Video S3), forming dense clusters 10–50 μm below the collagen-rich surface of the lung. eYFP-bright cells within the lung tissue on day 3 were more highly motile than remaining DCs on day 1 (Figure 2D). The sphericity of the eYFP-dim cells remaining in the lung on day 1 was significantly higher than eYFP^+^ cells before infection or on day 3 after infection (Figure 2E), consistent with alveolar macrophages remaining in the lung while eYFP-bright DCs migrate to the lymph node and then begin to return by day 3. The re-population of CD11c^+^ cells in the lung at this stage of influenza infection is carried out primarily by CD11c^+^CD11b^+^ inflammatory-type DCs [11]. No eGFP^+^ T cells were apparent in the lung before infection or during phase 1.

In the lymph node prior to infection, the resident, sessile DCs in the lymph node formed a network of cells that actively extended processes (Video S4, left; T cells  =  white tracks, DCs  =  blue tracks), as previously described [14]. Corresponding to the loss of lung DCs, eYFP^+^ DCs filled structures that connected to the subcapsular sinus space in the mediastinal lymph node on day 1, and that closely resembled afferent lymphatic sinuses in size, shape, and location (Figure S2A). Within 1 day of influenza infection, the arriving DCs migrated among resident DCs in the draining lymph node (Figure S2B; Video S4, center). To analyze DC behavior further, we divided DCs into two populations according to mean velocity (>6 μm/min  =  motile DCs, <6 μm/min  =  sessile DCs) and defined their motility within the lymph node. The percentage of motile resident DCs in CD11c-eYFP animals was identical to that previously reported in lymph nodes (4.7±1.4%) [14]. On day 1 following infection, the percentage of highly motile DCs in the lymph node increased significantly (25.2±3.6%) and remained elevated on day 3 (19.0±4.2%) relative to uninfected control lymph nodes (Figure S2C). Motile DCs may include resident DCs that are induced to migrate [21], as well as DCs originating from the lung. Highly motile DCs were tracked by hand; small, T cell-sized eYFP^+^ cells were excluded from analysis due to the possibility of an occasional CD8^+^CD11c^+^ T cell in the draining lymph node (<3% of CD8^+^ T cell population) [22]. Sessile control, day 1 and day 3 DCs were significantly larger than the motile DCs in the lymph node (Figure S2D). Motile DCs exhibited a myriad of behaviors within the lymph node, organizing into clusters (Figure S3E), and interacting extensively with sessile DCs (Figure S3F), potentially delivering antigen to resident DCs for T cell priming [23], [24], [25]. In summary, our imaging demonstrates a loss of lung CD11c^+^CD103^+^ DCs that corresponded to an influx of highly active DCs into the mediastinal lymph node on day 1 after virus infection, consistent with previous reports of rapid exit of lung-resident CD11c^+^CD11b^−^CD103^+^ DCs followed by arrival in the draining lymph node post-influenza infection [16], [26].

Under control conditions and during the first three days following infection, both eGFP^+^ CD8^+^ T cells and eYFP^+^ DCs were present in the mediastinal lymph node, consistent with previous studies demonstrating that the naïve CD8^+^ T cell population is sequestered, expands, and gains effector capabilities prior to infiltration of the lung [17], [27]. Control and day 1 CD8^+^ T cells were highly motile, while day 3 T cells had more confined movement (Video S4, white tracks, Figure 2F). Average T cell velocities were similar to control values on day 1, and by day 3 T cell velocities significantly decreased (Figure 2G). Because antigen transfer from migratory peripheral DCs to resident network DCs may occur prior to CD8^+^ T cell activation [24], and robust DC migration within the lymph node could serve as a mechanism for antigen distribution rather than T cell priming [14], [28], we separated CD8^+^ T cell and eYFP-DC interactions into two groups for analysis: T cell interactions with sessile DCs and T cell interactions with motile DC contacts. On day 1 following infection, contact durations between T cells and sessile DCs were already prolonged compared to control T-motile DC interactions, whereas on day 1, T-motile DC contacts remained brief (Figure 2H, Video S4). By day 3, CD8^+^ T cell contacts with sessile DCs were further prolonged, and were longer than T-motile DC contacts on day 3 (Figure 2H). These results indicate that T cell contacts are significantly more stable with sessile than with motile DCs. Despite little change in velocity and only a slight increase in contact duration, the average volume of T cells on day 1 was elevated relative to controls, an indication that T cells had encountered activation signals 24 h after infection, and by day 3 T cell volume was significantly higher than control and on day 1 (Figure 2I).

The changes in T cell size and velocity apparent by day 1 indicated that T cells had begun to respond to exogenous antigen, so we further examined T cell movement and applied directional persistence analysis [29] to determine if motility patterns were altered at this time (Figure 2J). Briefly, directional persistence describes the linearity of cell movement from its point of origin; a value of 1 would represent a straight line away from the point of origin, 0 neutral movement, and -1 movement directly towards the track origin. Control and day 1 CD8^+^ T cells exhibited similar mean directional persistence. However, unlike control cells, the directional persistence and velocity of individual cells were negatively correlated on day 1. This indicates that faster moving T cells (up to 25 μm/min) on day 1 post-infection tended to move in a more tortuous path relative to their origin, while slower moving cells demonstrated higher directional persistence. Increased CD8^+^ T cell confinement was previously reported to occur in an antigen-independent manner in the presence of activated DCs [30]. On day 3, T cells exhibited the highest mean directional persistence of all cell populations imaged in the lymph node and the lowest mean velocity (Figure 2G, J), indicating that day 3 T cells move slowly and along straighter paths.

On day 3 in the lymph node, CD8^+^ T cell division is a prominent feature of T cell priming. The rate of observed cell divisions was estimated to be ∼1,700/hr/μl, the maximum division rate recorded in this study. In contrast to previous imaging studies in which both CD4 and CD8^+^ T cell division was seen after detachment from antigen presenting DCs [30], [31], we found that CD8^+^ T cell division usually occurred while T cells maintained contact with a sessile eYFP^+^ APC. The observed T cell divisions fell into three categories: 1) both daughter cells maintained contact with the APC (Figure 2K, Video S5, left); 2) polarized division where only one daughter cell remained in contact with the APC (Figure S3A, Video S5, center); and 3) the T cell moves off the APC and then divided without visible APC contact (Figure S3B; Video S5, right). In half of the observed T cell divisions (50%) both daughter cells maintained contact with the APC; an equal number were polarized or APC-independent (25% each; Figure S3C). Interestingly, T cell division while in contact with an APC was relegated to T cell contacts with sessile, eYFP-dim APCs (Figure S3D). Prior to division, T cells moved in a directional manner towards the APC on which division occurred (Figure S3E). T cells, just prior to division, demonstrated significantly higher velocity and directional persistence relative to daughter cells after detachment from DCs and relative to total pooled day 3 CD8^+^ T cells (Figure S3F, G; *c.f.* Fig. 2G). This result is similar to previous reports of chemokine-guided CD8^+^ T cell movement toward an APC, during activation and generation of memory T cells [32].

### Phase 2 (Days 6–8): viral clearance; DCs repopulate the lung, effector CD8^+^ T cells migrate to the periphery

**Figure 3:**
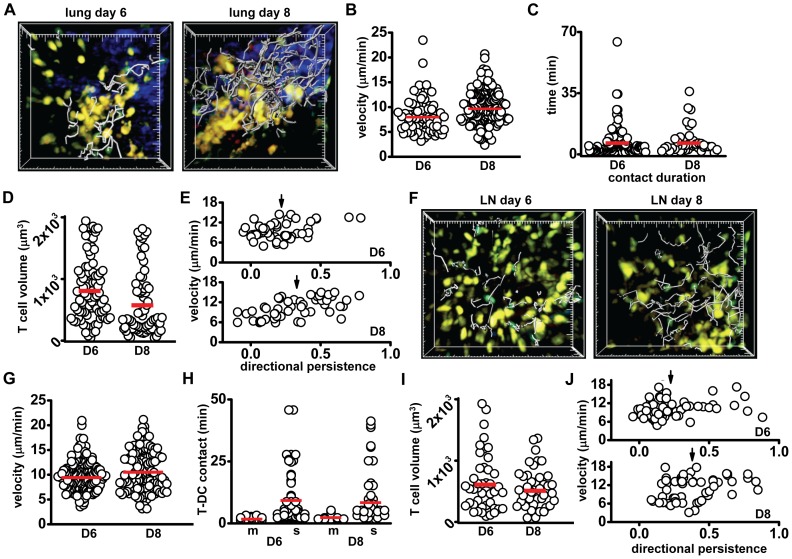
T and DC behavior in the lung and lymph node during the Clearance phase. (A) Images of T cells (green with grey tracks), shown with yellow DCs in the lung on day 6 and day 8 (16.5 min of T cell movement tracked, large tick marks  = 20 µm). (B) T cell velocities on days 6 and 8 (8.2±0.4 and 9.9±0.22 µm/min, respectively, p<0.01). (C) T-DC contact durations in the lung on days 6 and 8 (8.6±1.3 and 7.4±1.2 min, respectively, p = 0.86). (D) T cell volumes in the lung on days 6 and 8 (834±56 and 604±63 µm^3^, respectively, p<0.01). (E) Scatter plot of lung T cell velocity against directional persistence for day 6 (top; dp  = 0.22±0.02) and day 8 (bottom: dp  = 0.30±0.03; p = 0.02). Day 8 T cell dp and velocity are positively correlated (PCC  = 0.6, p<0. 01). (F) Images and tracks (grey) of lymph node T cells (green), shown with yellow DCs on day 6 and day 8. Tracks represent 16.5 min (large tick marks  = 20 µm). (G) T cell velocities in the lymph node on day 6 (10±0.21 μm/min) and day 8 (10.8±0.31 µm/min, p = 0.08; p<0.01 compared to day 3 for both, *c.f.* Fig. 2G). (H) T cell contact durations with motile DCs (m) remains low for day 6 (2.1±0.2 min) and day 8 (2.7±0.4 min). Both day 6 (9.7±1.3 min) and day 8 (8.8±1.3 min, p = 0.5) T cell contact with sessile DCs (s) is only slightly shorter than at day 3, but significantly higher than control. (I) T cell volume in the lymph node remains enlarged, and is not significantly different between day 6 (634±70 µm^3^) and day 8 (540±45 µm^3^, p = 0.8). (J) Directional persistence of CD8^+^ T cells in the lymph node on day 6 (dp  = 0.22±0.2) and day 8 (dp  = 0.38±0.02) plotted against velocity demonstrates a significant difference in directional persistence (p<0.01). Data from each tissue were pooled from 3–4 separate experiments; each individual dot represents the behavior of a single cell, bars represent the mean.

Consistent with previous descriptions of T cells arriving in the lung following influenza virus infection [33], CD8^+^ T cells were readily visible in the deep lung in close association with clusters of eYFP^+^ DCs on day 6 (Figure 3A, Video S6, left). On day 8, T cells appeared to be more numerous and migrated more rapidly than day 6 T cells (Figure 3A, B; Video S6, right). T-DC contact durations in the lung were not statistically different between day 6 and day 8 (Figure 3C). The percentages of motile DCs found in the lung tissue were not significantly different between days 3, 6 and 8 (all below 10%). T cells imaged on day 8 in the lung were significantly smaller than on day 6 (Figure 3D). Despite previous reports of T cell division in the lung after viral infection [33], [34], we failed to observe any examples of T cell division near DC clusters at the surface or in the parenchyma, possibly because cell division may occur earlier than day 6 or in the upper airways (not imaged in this study). Although day 6 and 8 T cells exhibit similar velocities, day 6 T cells had lower directional persistence, indicating a higher level of confinement (Figure 3E). Furthermore, day 8 lung T cells demonstrated a positive correlation between directional persistence and velocity, indicating that the fastest moving T cells were less confined. In summary, CD8^+^ T cells prior to viral clearance (day 6) have slower velocity, are larger in volume and have more confined motility than T cells imaged just after viral clearance on day 8.

Markedly fewer T cells were visible in the lymph node on days 6 and 8 as compared to day 3 (Figure 3F, Video S7). This matches the time when mature effector cells exit from the lymph node and T cells arrive in the peripheral tissue [27]. The mean velocity of T cells on day 6 and 8 was significantly faster than on day 3 (Figure 3G). As observed on days 1 and 3, T cell contacts with sessile DCs were longer than with motile DCs on both days 6 and 8 (Figure 3H). The percentage of motile DCs per imaging volume decreased significantly on day 6, relative to days 1 and 3, matching control numbers for motile DCs (4.4% and 4.7%, respectively, p = 0.8). However, the percentage of motile DCs increased again on day 8 (11.5%, p = 0.03); this increase may represent a second wave of CD11b^+^ DCs from the lung, consistent with previous data from influenza infection and skin inflammation [24], [35]. Overall, T cell volume in the lymph node on days 6 and 8 decreased relative to T cell volume on day 3, but was stable between day 6 and day 8 (Figure 3I). T cells on day 6 in the lymph node had lower directional persistence than on day 8 (Figure 3J).

In summary, relative to day 3 T cells, CD8^+^ T cells in the lymph node on days 6 and 8, exhibited decreased contact duration with sessile DCs, smaller cell volume, and increased overall velocity. On day 8 T cells demonstrated an increase in directional persistence relative to day 6 in the presence of a significantly higher number of motile DCs. In addition, on day 8 a rare T cell division was seen (Video S7, right); however, no proliferation was visualized on day 6 in the lymph node. In this phase, T cell proliferation in the lymph node is minimal, the number of visible T cells decreases, the percent motile DCs decreases, and activated T cells have entered the lung tissue. Therefore, Phase 2 represents a shift of T cell activity to the lungs, corresponding to viral clearance.

### Phase 3 (Days 10 and 14): maintenance, decreasing T cell numbers in the lung

**Figure 4:**
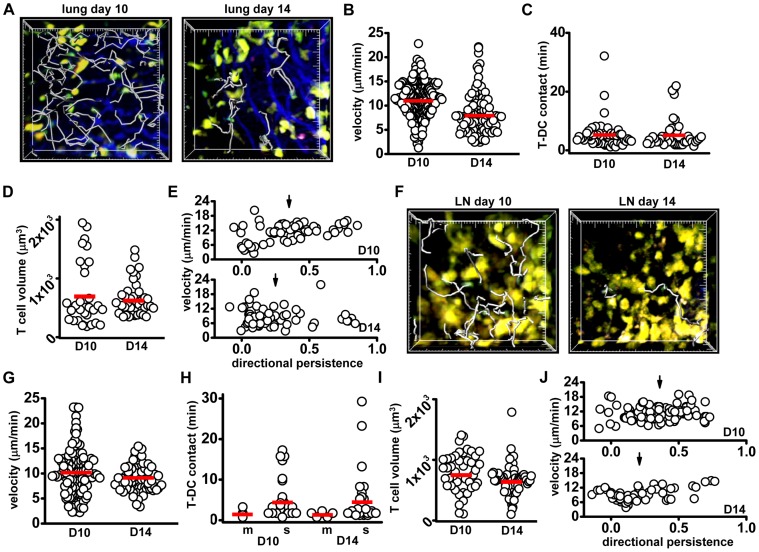
T and DC behavior in the lung and lymph node during the Maintenance phase. (A) Images of DCs (yellow) and T cells (green) in the lung 10 and 14 days after influenza infection; tracks (grey) represent 16.5 min of T cell movement, large tick marks  = 20 μm. (B) T cell velocities in the lung on day 10 (11.2±0.22 µm/min) and day 14 (8.3±0.4 µm/min, p<0.01). (C) T-DC contact durations in the lung on day 10 (5.8±0.9 min) and day 14 (5.7±0.8 min; p = 0.5, not significantly different). (D) T cell volumes in the lung on day 10 (721±98 µm^3^) and day 14 (652±45 µm^3^, p = 0.5, ns). (E) T cell directional persistence in the lung (plotted against velocity) on day 10 (0.35±0.03) and day 14 (0.25±0.03, p = 0.01). (F) Images of T cells in the lymph node on day 10 and day 14. Tracks (grey) represent 16.5 min, large tick marks  = 20 μm. (G) T cell track velocities in the lymph node on day 10 (10.0±0.4 µm/min) and day 14 (9.0±0.3 µm/min; p = 0.03). (H) T cell contact durations with motile DCs on day 10 (1.8±0.6 min) and day 14 (1.7±0.2 min, p = 0.7, ns); and with sessile DCs on day 10 (4.8±1 min) and day 1 (4.7±0.8 min, p = 0.35, ns). (I) T cell volumes in the lymph node on day 10 (796±48 µm^3^) and day 14 (669±32 µm^3^, p = 0.02). (J) Directional persistence plotted against velocity for day 10 (dp  = 0.36±0.02) and day 14 (dp  = 0.21±0.03, p<0.01). Velocity correlates with directional persistence in day 14 T cells (PCC  = 0.47, p<0.01). Data from each tissue were pooled from 3-4 separate experiments; each individual dot represents the behavior of a single cell, bars represent the mean.

In the lung on days 10 and 14 post-infection, T cells were less confined to clusters of DCs, and T-DC interaction times were shorter than during phase 2 (Figure 4A, Video S8). Near the surface of the lung, eYFP-bright DCs were distributed along collagen fibers, where highly motile T cells crawling along collagen fibers made brief contacts (Figure S4A, B; Video S8). Clusters of DCs were occasionally visible in the inner lung tissue, and around larger airways in slice preparations, although fewer T cells were found in these regions relative to the lung surface (Figure S4C). T cells near the lung surface on day 10 had significantly higher average velocities than on day 8, but by day 14 lung T cell velocities decreased relative to day 10 (Figure 4B). Despite the decrease in average velocity, on day 14 T-DC contact durations remained brief (Figure 4C). Also, on both day 10 and 14, T cells were seen to briefly enter alveolar spaces (Figure S4D, E; Video S9). At these time-points the percentage of motile eYFP-bright DCs in the lung tissue was low (<5%), matching control numbers for percent motile lung DCs, and the volumes of lung T cells remained low relative to day 6 post-infection (Figure 4D). T cell directional persistence on day 10 increased relative to day 8, but decreased again on day 14 (Figure 4E). In summary, lower velocities, increased confinement, and fewer T cells per imaging volume on day 14 indicated a transition from the T cell tissue-scanning behavior seen on day 10, to the contraction of the effector T cell population on day 14.

In the lymph node on days 10 and 14, fewer T cells were visible compared to days 6 and 8 (Figure 4F, Video S10). Day 10 T cell velocity was not different from days 6 and 8; however, day 14 T cell velocity decreased relative to day 10 (Figure 4G). In addition, day 10 T-sessile DC contact times in the lymph node decreased significantly relative to day 8 and remained brief through day 14 (Figure 4H). On day 14, A small percentage of T cells on days 10 (14%) and 14 (9%) demonstrated an extended DC contact duration, (>10 min, up to 30 min), which may represent cells continuing to receive antigen stimulation. As seen on day 8, the percent of motile DCs found in the draining lymph node on day 10 (11.1±1.4%) is higher than the percent of motile DCs found in control lymph nodes, but decreased significantly by day 14 (2.1±0.5%, p<0.01). T cell volume also decreased between day 10 and day 14 (Figure 4I). Motility of T cells on day 10 and 14 differed only in directional persistence; day 10 T cells had a significantly higher mean directional persistence than day 14 T cells (Figure 4J), indicative of less overall confinement and perhaps greater tissue scanning capabilities of day 10 T cells, relative to day 14 T cells.

### S1P receptor agonist treatment delays T cell activation during influenza infection

**Figure 5:**
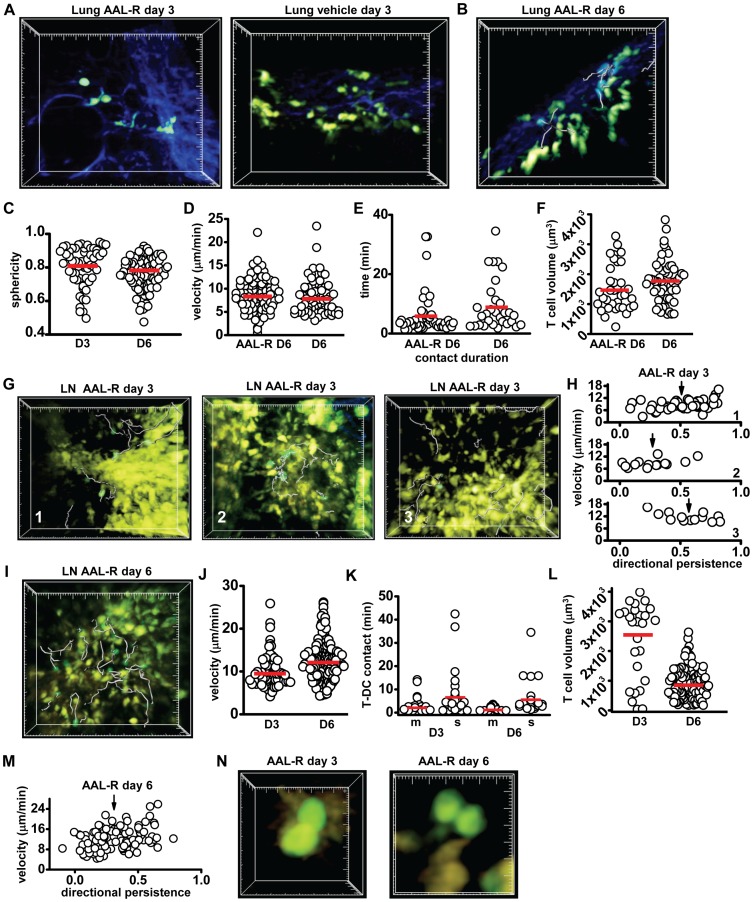
AAL-R treatment modifies T cell and DC dynamics. (A) Image of the deep lung from an AAL-R-treated animal on day 3 (left), compared to vehicle-treated lung on day 3 (right, large tick marks  = 20 µm). (B) Image of the deep lung on day 6 and T cell tracks (25 min tracks, grey; large tick marks  = 20 µm). (C) Dendritic cell sphericity in the lung on day 3 (0.8±0.02) and day 6 (0.77±0.01, p = 0.03). (D) Velocity of T cells on day 6 in AAL-R treated (8.7±0.4 µm/min) animals and in vehicle-treated lungs (8.2±0.4 µm/min; p = 0.4, ns). (E) Contact duration of T cells with DCs in the lung on day 6 in AAL-R treated animals (6.2±1 µm/min) and in vehicle-treated lung (9.7±1.6 µm/min, p = 0.04). (F) T cell volumes in AAL-R-treated lung (1520±130 µm^3^) and in vehicle-treated lung (1823±114 µm^3^; p = 0.13). (G) T cell tracks (grey) in three regions in the lymph node defined by dendritic cell patterns of distribution: 1) outlined lymphatics (T cell tracks  = 29.5 min); 2) large clusters of DCs (T cell tracks  = 1∶29 hr:min); and 3) even distribution (T cell tracks  = 29.5 min). All large tick marks  = 20 µm. (H) T cell velocity plotted against directional persistence in these three regions (region 1, T cell dp  = 0.51±0.03; region 2, dp  = 0.27±0.05; region 3, dp  = 0.57±0.05; p<0.01 for region 2 compared to both regions 1 and 3). T cell velocity and directional persistence show a negative correlation in region 3 (PCC  = −0.6, p = 0.03) and a positive correlation in regions 1 and 2 (PCC  = 0.5, p = 0.0005, and 0.53 p = 0.05, respectively). (I) T cell tracks (grey) in the lymph node on day 6 after AAL-R treatment (8 min tracks). (J) T cell velocities in the lymph node of AAL-R-treated animals on day 6 (12.5±0.32 µm/min) and day 3 (9.9±0.38 µm/min, p<0.01). (K) T-DC contact durations with motile DCs (m, 3.1±0.7 min) or with sessile DCs (s, 7.4±2 min, p = 0.02). T cell contact durations with sessile DCs on day 3 and day 6 (7.4±2 and 6.1±1.5 min, respectively, p = 0.9). T-DC contact with motile DCs on day 6 is significantly lower than contact with sessile DCs on day 6 (1.7±0.2 and 6.1±1.5 min, respectively, p<0.01). (L) T cell volumes in the lymph node on day 3 and day 6 (2600±252 and 902±50 µm^3^, respectively, p<0.01). (M) T cell velocity plotted against directional persistence in lymph node on day 6 of AAL-R-treated animals. (N) T cell division on day 3 and day 6 occurs primarily while in contact with a sessile eYFP^+^ DC. Data from each tissue were pooled from 3–4 separate experiments; each individual dot represents the behavior of a single cell, bars represent the mean.

S1P agonist treatment suppresses the development of cytokine storm and associated pathology characteristic of severe influenza infection, without compromising the kinetics of viral clearance [7]. Here we investigated the effects of the S1P agonist AAL-R on the progression of T cell responses and dendritic cell behavior during influenza infection. Shortly after infection, animals were treated with a single dose of AAL-R (i.t.) and tissues were harvested at days 3 and 6 for imaging. In contrast to infected animals without AAL-R treatment, there was little to no re-population of YFP-bright DCs in the lung on day 3 (Figure 5A). To ensure deep-lung clusters were not overlooked, slice preparations of the treated lung tissues were made. No DC clusters were evident. By day 6, some clusters of DCs were visible just below the surface of the lung; however, these DC clusters were more diffuse than the day 6 DC clusters in untreated animals (Figure 5B, Video S11). In AAL-R-treated animals on day 3, the sphericity of the remaining lung eYFP^+^ cells was similar to that on day 1 APC in untreated animals and was higher than cell sphericity in lung from both vehicle-treated and untreated animals, indicating that DCs had not yet begun to repopulate the lungs (Figure 5C). T cells in lungs of AAL-R-treated animals on day 6 were found in the vicinity of diffuse DC clusters and exhibited similar motility as T cells in lung tissue from vehicle-treated control animals (Figure 5D). However, the contact duration of T cells with local DCs in the lung was significantly shorter in treated animals relative to control (Figure 5E), indicative of a milder response. Finally, T cells in the lungs of AAL-R treated animals had similar cell volumes relative to vehicle-treated animals (Figure 5F).

Fewer T cells were visible in the lymph node of AAL-R treated animals on day 3 after infection as compared to control day 3 lymph nodes (Figure 5G), indicating a delay or inhibition of cell proliferation. The percentage of motile DCs in the lymph nodes of AAL-R-treated animals relative to untreated animals on day 3 was similar (17±3.3% and 19±4.4%, respectively). Despite a similar percentage of motile DCs, the distribution of DCs in the lymph node was noticeably different between day 3 untreated and day 3 AAL-R-treated animals. Three regions within the lymph node were identified in the lymph nodes of AAL-R-treated animals characterized by distinct distributions of dendritic cells: 1) afferent lymphatics were filled with DCs in several regions; 2) large clusters in which active DCs grouped together; and 3) diffuse zones that contained independently motile DCs which were most similar to day 3 control lymph nodes (Figure 5G, Video S12, left, right, and center). T cell behavior was measurably different in each of these regions. First, T cells did not enter the afferent lymphatic areas outlined by DCs, but crawled around and over these areas with relatively slow mean velocity and high mean directional persistence (Figure 5G and H, Video S12, region 1, left). Second, T cells that entered clusters of DCs moved with significantly lower directional persistence than DCs near the lymphatic sinus, but with similar overall mean velocities (Figure 5G, H, region 2, center). Third, T cells in regions of diffusely organized DCs were found to have a higher track velocity than either lymphatic-sampling or cluster-associated T cells, and moved with high directional persistence (Figure 5H). Similar to day 1 T cells in untreated animals, T cells in regions of relatively diffuse DCs demonstrated a strong negative correlation between velocity and directional persistence; therefore, slower cells demonstrated higher directional persistence, while faster cells embarked on a more tortuous path, similar to day 1 T cells in untreated animals. Although T cell division was seen on day 3, it was relatively rare (∼120/hr/μl) compared to control animals, and only occurred in the regions of diffuse DCs that were typically deeper in the T cell zone of the lymph node (100–150 μm below the capsule). Therefore, in general, T cells in AAL-R-treated animals on day 3 demonstrated behavior that appeared to be a mix of day 1 and day 3 control T cell behavior.

DC clusters and lymphatic DCs had dispersed in the lymph node of AAL-R-treated animals by day 6, although T cells were far more abundant than in control animals (Figure 5I, Video S13), consistent with a delay in proliferation and inhibition of egress. T cell velocities in the lymph nodes of AAL-R-treated animals were on average higher than the pooled day 3 control T cell velocities (Figure 5J, *c.f.* Fig. 2G). CD8^+^ T cell division in AAL-R treated mice occurred more often on day 6 (Video S13, dividing cells circled) than on day 3, at an estimated rate of 1,100/hr/μl and only slightly less than T cells in untreated animals on day 3 (∼1,700/hr/μl). In general, day 6 T cells had a higher average velocity, and slightly shorter T-DC contact times (Figure 5K) and a lower mean volume (Figure 5L), than day 3 T cells in AAL-R treated animals. In addition, day 6 T cells had a lower overall directional persistence when compared to pooled day 3 AAL-R treated T cells and a positive correlation between velocity and directional persistence (Figure 5M). In line with our observations of T cell behavior during division, on day 3 and day 6 in AAL-R treated animals T cell division occurred while in contact with a sessile APC (Figure 5N). In summary, AAL-R treatment delayed CD8^+^ T cell division, an effect that may be caused by reduced antigen delivery by DCs to the lymph node.

## Discussion

**Figure 6:**
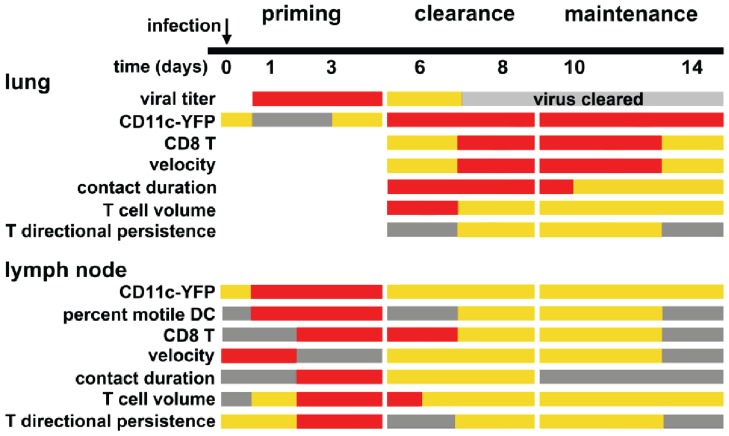
Summary of T cell and DC behavior over 14 days of influenza infection. Relative magnitudes of cell numbers (grey  =  low abundance or no cells detected, orange  =  intermediate, and red  =  high), viral titre (grey  =  low or no virus detected, orange  =  intermediate, and red  =  high), and relative values of imaging parameters during 3 phases.

The dynamic ebb and flow of dendritic cell and T cell migration and T-DC interactions within each environment illuminate the intimate association between the draining lymph node and peripheral tissues during an effector response to viral infection. Here we describe the kinetics of CD8^+^ T cell and dendritic cell interactions over a 14 day time course of an immune response to influenza A infection. A heat map of T cell and dendritic cell activity in the lung and lymph node over the course of 14 days after infection illustrates three overlapping phases of T cell and DC response to influenza infection: 1) Priming; 2) Clearance; and 3) Maintenance (Figure 6). During the priming phase, the adaptive response is initiated following a long-range migration by DCs bringing antigen to the lymph node. Flu-specific T cells recognize antigen and begin to proliferate. During the clearance phase, activated CD8^+^ T cells (CTLs) repopulate the lung in a reciprocal long-range migration from the draining lymph node to the lung. CD8^+^ T cells then recognize and kill virus-infected cells. In the maintenance phase, T cells continue to scan the lung, but in diminished numbers. Below, we provide a summary of T cell and DC dynamics for each of the phases and, when relevant, compare cellular dynamics of influenza infection with T cell and DC responses to immunization by antigen with adjuvant.

### Phase 1: DC mobilization and T-cell priming

The first response of the adaptive immune system to influenza infection is the initiation of a long-range migration of antigen-bearing CD11c^hi^, CD11b^low/neg^, CD103^+^ lung-resident dendritic cells to the mediastinal lymph node, followed by T cell priming (Fig. 2, [36]). Murine lung tissue is patrolled by two subsets of conventional CD11c^+^ DCs, distinguished by CD11b and CD103 expression. CD103^+^CD11b^-^ DCs participate in viral antigen cross-presentation and antigen transfer through MHC-I antigen-bearing DC membrane exchange [16], [23], [24], [25], [26], [37], [38]. We show that CD103^+^CD11b^-^ DCs are enriched near the surface of the lung as demonstrated by flow cytometry of micro-dissected tissue. Two-photon imaging of brightly-fluorescent eYFP^+^ DCs revealed that this DC population is readily imaged in control animals but is absent near the lung surface 24 h after viral infection, coinciding with the arrival of highly motile DCs in the draining lymph node. At 24 h, only a few eYFP-dim cells remain in the lung, mostly located in and around alveolar spaces. The remaining eYFP-dim cells consist of a mixture of APCs that likely includes alveolar macrophages, the rare plasmacytoid DCs, and CD103^-^CD11b^hi^ DCs that become activated upon viral infection, but stay behind in lung tissues supporting the early immune response to viral infection [39], [40], [41].

T cells become activated in the draining lymph node and begin to divide during Phase 1. The behavior of T cells in the lymph node on day 1 is marked early on by active migration but increased confinement as T cells encounter antigen-bearing DCs, and is similar to the first stage of the T cell response to specific antigen [30], [31]. However, T cell swarming behavior, as previously described [30], [31], did not occur on either day 1 or day 3 of our imaging experiments. An absence of swarming behavior could be for one of several reasons. First, T cell swarm formation may occur transiently at an intermediate time point between days 1 and 3. Second, in our experiments the numbers of adoptively transferred antigen-specific cells were much smaller (∼1% to 0.5% of antigen-specific cells transferred in previous studies), so that fewer visible cells were available to identify a swarm. Third, DCs from the lung enter and migrate rapidly throughout the lymph node. This rapid migration may promote extensive antigen distribution throughout the sessile DC network, producing several available sites for T cells to encounter antigen. Therefore, during infection T cells may not have to seek a rare antigen-bearing DC, but instead, antigen distribution throughout the DC network by highly motile peripheral DCs may allow for rapid cognate antigen identification within the draining lymph node.

Unlike previous reports of T cell division in the lymph node [30], [31], our imaging demonstrated that T cells preferentially divide while in contact with a sessile eYFP^+^ APC. These sessile APCs may represent either a lymph node-resident population of DCs, macrophages, or recent migrants that have settled into the sessile network. Antigen presentation by both resident and migratory DCs is important for effective CTL responses, and recent DC emigrants are an essential source of antigen [25]. It is likely that T-DC contact during T cell division was not reported by other studies [30], [31] because only the antigen-presenting cells from the periphery were labeled. Interestingly, prior to initiating contact with the DC that supports division, T cells moved in a directional manner towards the target DC, similar to previous reports of APCs directing local CD8^+^ motility prior to T-DC contact [32]. Behavior during T cell division was heterogeneous; some cells divided in a polarized manner with only one daughter cell maintaining contact with the local DC and in other divisions both daughter cells maintained contact with the local DC. Asymmetric and symmetric DC-daughter T cell contact (or lack of T-DC contact) during division likely represents an early step in T cell programming by local DCs during the developing immune response that produces the heterogeneity of virus-specific CD8^+^ T cell lineages [42], [43], [44].

### Phase 2: Viral clearance

Lung imaging revealed that T cells begin to arrive on day 6 and encounter DCs that had already arrived to repopulate the lung. T-DC interactions on day 6, prior to viral clearance, are characterized by the longest lasting interactions of all time points examined. These extended interactions likely represent the reactivation of effector CD8^+^ T cells in the periphery, an early step in the initiation of effector function that may allow for local APC titration of the T cell response [40]. Two days later, coinciding with viral clearance in the lung, T cell velocity moderately increases as T-DC interactions slightly decrease together with decreases in T cell volume and directional persistence. The decrease in directional persistence, despite higher velocities and shorter contact times, indicates that CD8^+^ T cells demonstrate higher overall confinement, which may be due to interactions with and killing of virus-infected cells. Contrary to other reports of effector cell division at the site of viral infection in the brain and lung [33], [45], T cell division was not seen in the lung on day 6, but may have occurred upon initial T cell arrival in the lungs (day 4–5) [33], or in regions such as upper airways that were not imaged in this study. Instead, enlarged T cells were observed crawling on collagen fibers and frequently associated with local APCs, behavior reminiscent of effector T cells in skin during a DTH response [46].

In the lymph node, T cell division was not seen on day 6, but did occur rarely on day 8. It is possible that a rare division event on day 6 was missed in our imaging, or that a second wave of antigen-bearing DCs, (apparent in a significant increase in motile DCs on day 8 and 10) from the periphery supports additional T cell division on day 8. In support of this hypothesis, T cell velocities and directional persistence markedly decreased on day 6 only to recover on day 8, mimicking day 1 T cells that are just encountering peripheral DCs. In line with our results, previous studies have reported that after virus clearance, antigen-bearing DCs migrate from the lung to the draining lymph node where they contribute to the maintenance of antigen-specific CD8^+^ memory T cells [47], [48].

### Phase 3: Maintenance

On days 10 and 14 (2 and 6 days after complete clearance of detectable virus in the lung), fewer CD8^+^ T cells were visible in the lung, and these cells associated less frequently with DCs. Instead T cells travel along collagen fibers and actively sample alveolar spaces. By day 14, T cell motility decreased relative to day 10 and it was difficult to find cells in the tissue, although they may have dispersed more evenly throughout the lung (no longer associated with areas of high DC density). Fewer visible T cells corresponds to the contraction of the effector T cell population. Dendritic cells in the lung tissue after viral clearance are reported to form tertiary lymphoid tissues that are maintained for up to a month post infection [49]. Near the lung surface we found that eYFP^+^ DCs were highly dispersed, and although some clusters remained near larger airways, T cells did not engage those DCs for an extended period of time. These DC-independent T cells moving throughout the lung tissue are likely full effector T cells capable of killing a virus-infected cell. Therefore, T cell scanning behavior within the infected tissue may represent a ‘seek and destroy mode’ to remove any residual infected cells and maintain the state of viral clearance.

In the lymph node, cell volume, velocity and number of visible cells significantly decreased during the maintenance phase. Although contact duration with sessile DCs decreased on average, an occasional T cell maintained extended contact (>10 min) with a sessile DC. These interactions may be due to residual antigen presentation which plays a significant role in the development of memory CD4^+^ T cells after influenza infection [50], but may not always be necessary for CD8^+^ T cell memory development [51]. In general, however, T cell reduction in size and numbers on days 10 and 14 overlap with the CD8^+^ T cell contraction during which 95% of effector cells undergo apoptosis [52].

T cell velocities increase in the lung just after viral clearance, then decrease as T cell numbers and scanning behavior in both the lung and lymph node is diminished by day 14. In the lymph node the number of visible T cells continues to decrease relative to the peak seen on day 3, and T cell size and velocity are lower as the immune response wanes.

### Sphingosine 1-phosphate receptor agonist treatment

Intra-tracheal treatment with sphingosine 1-phosphate agonists AAL-R and CYM-5442 after influenza infection suppresses DC migration to the draining lymph node, inhibits DC maturation, reduces serum levels of pro-inflammatory cytokines, and perhaps most relevant for therapeutic potential, AAL-R demonstrates superior protection from mortality over neuraminidase inhibitor after challenge with a lethal dose of influenza [1], [2], [3], [7], [8], [53]. Furthermore, treatment with S1P agonist AAL-R reduces CD8^+^ T cell accumulation in the lung tissue after infection [53]. Surprisingly, suppression of the adaptive effector cell response does not alter kinetics of viral clearance [8], [53].

Our data demonstrate that DC infiltration of the lymph node from the periphery, as well as T cell division, were delayed by a single dose of AAL-R. Although T cells did make some long-lived contacts with local DCs on day 3 in AAL-R-treated animals, they failed to divide. The majority of imaged T cell proliferation occurred on day 6 rather than day 3 after infection. The delay in peak T cell proliferation may be due not only to an apparent delay in DC entry but also to AAL-R suppression of lung DC maturation upon exposure to antigen. Treatment with AAL-R causes significant down-regulation of MHC class I and MHC class II as well as co-stimulatory ligand CD86 [7]. It is possible that an APC presenting only low levels of antigen and lacking full co-stimulatory capabilities could cause T cell enlargement seen on day 3 with AAL-R treatment without inducing T cell division. Interestingly, T cell accumulation in the lung at later time points (day 6) appears to be inhibited while T cells spend less time in contact with the local DC population in the lung. Therefore, in line with previous studies that demonstrated suppression of the effector T cell response to influenza upon AAL-R treatment, we find that the arrival of motile DCs and T cell division in the lymph node is significantly delayed on day 3 [2], [7]. Furthermore, T cells in the lung on day 6 interact less with local eYFP^+^ DCs which did not form large clusters but were instead diffuse and more difficult to find in the tissue.

In summary, this study characterizes the behavior of CD8 T cells and eYFP-CD11c DCs during the priming, clearance, and maintenance or contraction of an immune response to the pandemic flu virus H1N1. Our results also reveal important phase lags in T cell and DC dynamics induced by targeting S1P receptors in vivo and suggest that the timing of long-range migrations helps to determine the intensity of the immune response. We propose that selective disruption of cellular trafficking between lung and lymph node as a key target for therapies to prevent the cytokine storm that causes long-term lung damage. Finally, our quantitative imaging analysis provides the framework for future studies to evaluate potential therapies that target different phases of the response to infection. Imaging immune responses in the lung has only just begun to reveal the myriad of cellular interactions that occur in this organ.

## Materials and Methods

All procedures were in accordance with National Institutes of Health (NIH) guidelines and approved by Institutional Animal Care and Use Committees at the University of California, Irvine, and at The Scripps Research Institute.

### Virus infection and imaging model

The CD8^+^ T cells recognizing LCMV Gp33-41 (P14) originated from H-2b mice engineered to express the T cell receptor for Gp33-41 [18]. The TCR mice were crossed to transgenic mice expressing GFP under transcriptional control of β-actin [54]. GFP-TCR Gp33-41 CD8^+^ T cells were isolated from spleens of crossed mice by negative selection using a CD8^+^ T cell enrichment kit (STEMCELL technologies, Vancouver, Canada). The purity of GFP-TCR Gp33-41 cells was >98%. 5×10^4^ GFP-Gp33-41 CD8 T cells were adoptively transferred via tail vein injection into mice expressing eYFP under the CD11c promoter [14] 24 h prior to infection. Animals were infected i.t. with 1×10^5^ PFU of the recombinant A/WSN/33 mouse adapted influenza virus engineered to express Gp33-41 from lymphocytic choriomeningitis virus (LCMV) ARM3b that comprises the major immunodominant CD8^+^ T cell epitope for H-2b (H-2b D restricted) mice (Flu/LCMV) [53]. AAL-R was administered i.t. (0.2 mg/kg) as previously described [53] 30 min post infection.

Several preparations for lung imaging have been developed recently, including tissue slices [55], suction windows [15], [56], and timed acquisition [57] to minimize motion artifacts due to respiration. To image the immune response to viral infection in the lung we used a semi-intact lung preparation in which agrose-filled lungs were sliced at a distal site and placed in an imaging chamber slice-site down but otherwise intact. This allowed us to scan the tissue where we found the majority of T cells and T-DC interactions near the outer edges of the lung and image for extended periods at several sites in the same lung tissue. This preparation was novel in that imaging was not performed within several millimeters of a slice site, but instead at the surface of the lung. 24 h post infection animals were sacrificed and lungs were filled with 10% low-melt agarose (Sigma Aldrich, St. Louis, MO USA) maintained at 37°C in a water bath. A total volume of ∼1 mL was used to inflate the lungs accounting for some loss in the trachea. Slow inflation of the lung was important to minimize trapping of air that obscures two photon laser penetration of tissue. Mediastinal lymph nodes were harvested from behind the lung after inflation and removal of the lung tissue. Tissue was intact for the majority of the imaging experiments, except for the separation of lung lobes for mounting in the imaging system. Tissue mounting onto unbreakable plastic cover slips (General Laboratory Supply, Pasadena TX, USA) was achieved using a small amount of VetBond^TM^ (3 M Corporation) on the underside of the lung lobe or mediastinal lymph node. Slice preparations were occasionally made (as noted in the text) using a vibratome to bisect a lung lobe horizontally to allow for access to internal tissues and deep alveolar spaces for imaging. Imaging in slice preparations was performed a minimum of 50 μm below the site of tissue bisection. Tissue was kept on ice prior to imaging and during imaging tissue was submerged in a bath of oxygen perfused RPMI maintained at 37°C and fed by peristaltic flow, as previously described [46]. To highlight blood vessels 655 Qdots (Invitrogen, USA) were injected i.v. 5 min prior to sacrifice.

### Imaging system

Two-photon imaging was performed using a previously described system [46] with a Chameleon Ultra II Ti:Sapphire laser (Coherent Inc. Santa Clara, CA USA). Laser excitation was tuned to 880–900 nm to maximize eGFP and eYFP fluorescence, and second harmonic generation that marks the collagen fibers. 510 and 540 nm dichroic mirrors (Semrock, Inc. Rochester, NY USA) were used in series to separate emission into blue (<510 nm), green (510–540 nm) and red (>540 nm) channels. 3D image stacks were acquired using Slidebook version 4.2 and 3D stacks were compiled in Metamorph version 6.1 (Molecular Devices, LLC USA). Depending upon depth of the z-stack, time-points were taken every 18 to 27 seconds.

### Image analysis, statistics, and equations

Imaging analysis was performed in Imaris version 7.1.1. Increased power to the blue-channel PMT and Gaussian filtering was applied to improve visual separation of eGFP and eYFP. All cell tracking, brightness, and volume measurements were performed and/or edited by hand to ensure accuracy. Dendritic cells that moved on average >6 μm/min were considered motile. DC brightness is denoted as eYFP-dim or eYFP-bright when imaged, and eYFP^lo^ or eYFP^hi^ when analyzed by flow cytometry. Although they are not typically bright enough to be visualized using 2-photon microscopy [14], some lymphocytes express CD11c and are therefore potentially labeled with eYFP in this mouse model. In final videos, cells were pseudocolored to enhance color separation between eYFP and eGFP. During analysis the rare potential lymphocyte was excluded. For example, any eYFP^+^ cell without obvious additional sampling or projections while crawling that was in the size-range of an activated T cell ∼10–12 μm in length was excluded. Data sets were tested for normality and either a two-sample t-test (normally distributed data sets) or non-parametic analysis by Mann-Whitney U –test (non-normal distribution) were applied to determine statistical significance. The Pearson correlation coefficient (PCC) represents the degree of correlation (either positive or negative, value range: -1 to 1), where 0.4–0.6 is generally interpreted as a moderate correlation, and ≥0.6 can be interpreted as a strong correlation [58]. All statistical analysis was performed in OriginPro version 8 (OriginLab Corp., Northampton, MA USA). Sphericity (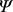) was calculated in Imaris using the following equation:
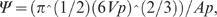
(where Vp  =  the volume of a particles, Ap  =  the surface area of the particle, and 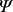 = 1 for a perfect sphere. Directional persistence [29] was calculated for every two steps (step 1, 2 and step 2, 3 etc.) for sequential 3D coordinates of cell position from the following equation:



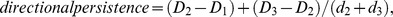
where D  =  displacement d  =  distance; value range of -1 to 1).

The number of cell divisions per hour per microliter was estimated by counting the number of cell divisions observed in a given imaging volume for the duration of the Video (typically 45 minutes to 1.5 hours). The number of divisions/hour/microliter were then averaged across at least 2 experiments (6–10 videos total). The number or n value given in the figure legends, unless otherwise stated refers to separate experiments.

### FACS analysis

Agarose-inflated lungs from wild type or CD11c-eYFP mice were harvested and micro-dissected under a 40x dissecting microscope, where ∼ 200 mm of the surface of the lungs was separated from the inner lung parenchyma. A single cell suspension of the tissue samples was made in ice cold 5% BSA and 2% FCS, stained with primary antibodies for 10 min prior to wash and fixation in 1% PFA (all listed reagents: Sigma Aldrich, St. Louis, MO USA). Antibodies used for labeling lung resident DCs harvested from either eYFP^+^ or wt lung tissue: anti-mouse CD11c-FITC, PE, or APC conjugated clone #N418, Armenian Hamster IgG FITC, PE or PE-Cy5 ITC, CD11b-PE clone #M1/70, Rat IgG2bκ-PE ITC (BD Biosciences, San Jose, CA), anti-mouse CD103-PerCP-Cy5.5 clone #2E7, Armenian Hamster IgG PerCP-Cy5.5 ITC, (BioLegend, San Diego CA). FACS data was acquired on a BD LSR II (BD Biosciences, San Jose CA, USA) and analyzed using FlowJo version 8.7.3.

## Supporting Information

Figure S1 **CD11c^hi^ DCs near the lung surface are CD103^+^.** (A) FACS analysis of the lung surface gated on a high forward scatter profile (left), revealing CD11c^hi^ and CD11c^lo^ populations. CD103 expression in these two populations (right). (B) Expression of CD103 in CD11c^hi^, relative to CD11c^lo^ cells on the surface of the lung (ratio  = 2.1±0.7). Data are representative of 3 separate experiments.
(TIF)

Figure S2 **Early DC infiltration and behavior in the lymph node.** (A) Compiled image of DCs in the lymph node on day 1 show eYFP^+^ cells outlining afferent lymphatic space. (B) Tracks of motile DCs deep in the lymph node on day 1 post-infection (46∶43 min:sec), large tick marks  = 10 μm. (C) Percent motile DCs (eYFP^+^) cells in the lymph node is significantly higher on day 1 (25.2±3.6%, p<0.01) and day 3 (19.0±4.2%) compared to the percent of motile DCs in the control lymph node (4.7±1.4%, p<0.01, both). (D) Dendritic cell volume v. track velocity plots show that cells with an average velocity >6 μm/min have significantly lower volume (closed circles) relative to eYFP^+^ DCs moving <6 μm/min (open circles). In control lymph nodes (volume motile  = 570±75 μm^3^, sessile  = 1209±287 μm^3^, p<0.01), day 1 (volume motile  = 715±68 μm^3^, sessile  = 1643±206 μm^3^, p<0.01) and day 3 (volume motile  = 803±140 μm^3^, sessile  = 2002±328 μm^3^, p<0.01). Mean values for each group are denoted by the red dot. (E) DCs exhibit several different behaviors on days 1 and 3 in the lymph node, here on day 3 DCs move together to form a sessile cluster. (F) A motile DC engages, and crawls on and around a sessile cluster of DCs, large tick marks  = 5 μm; track duration  = 36∶53 min:sec. Data were compiled from 4–6 separate experiments; each dot represents measurements taken from a single cell.
(TIF)

Figure S3 **Characteristics of dividing T cells in the lymph node on day 3.** (A) Images of a CD8^+^ T cell dividing in a polarized manner while in contact with a sessile DC. White arrows in the last frame point to the direction of movement taken by the daughter cells. (B) Images of a CD8^+^ T cell division while not in contact with a sessile DC. (C) Analysis of 20 examples of cell division in 3 separate lymph nodes, 3 days after influenza infection. Most cells divide while in contact with a sessile DC. (D) Brightness of DCs in contact with T cells leading to division, and alone (mean relative brightness  = 0.9±0.26) normalized to all DCs in the imaging volume (mean relative brightness ratio  = 2.4±0.1), where the dimmest visible cell  = 0; n = 3 separate experiments. (E) Time-lapse images of a CD8^+^ T cell on day 3. The cell makes a sharp turn and moves in a highly directional manner prior to division on a sessile DC; track duration  = 49∶32 min:sec. (F) T cell velocity prior to contacting DC and dividing (11.4±1.8 μm/min, n = 8 tracks) and daughter cell velocity after detachment from the DC (8.4±0.5 μm/min, n = 16 tracks, p = 0.04). (G) Analysis of T cell directional persistence (5–10 min) prior to contact with a DC on which division occurs. Counts represent the directional persistence of every two steps taken by the T cell. T cells showed high directional persistence (0.63±0.05, n = 8 cells), compared to both daughter T cell motility (n = 16 cells) after division (0.35±0.04, p<0.01), and pooled day 3 T cells (0.36±0.02, p<0.01, n = 4 separate experiments for all division data).
(TIF)

Figure S4 **T cell motility and behavior in deep lung parenchyma.** (A) T cell tracks (grey) in the lung on day 10 over 40 minutes of imaging demonstrate that T cells preferentially crawl along collagen fibrils embedded with eYFP^+^ DCs (left), represented in a space filling model where collagen fibrils are blue, APCs are gold and T cell tracks are grey (right; large ticks  = 10 µm). (B) Percent of time T cells in the lung spend in contact with a visible collagen fiber steadily increases between day 6 (51±7%) and day 14 (82±4%, p<0.01). (C) Cluster of DCs on day 10 in the lung were found deeper in the lung tissue (100 microns from the surface), than clusters of DCs imaged at earlier time points. (D) Close up of collagen bands (blue) supporting alveolar sacs in the deep lung (and outlining alveolar space) and T cells (green) that occasionally enter the alveolar space. (E) A series of images where in both a T cell (green, panels 0∶28 to 13∶46 min), a motile DC (yellow, panels 17∶05 to 37∶32 min), and alveolar macrophage (yellow, panels 20∶54 to 40∶51 min) probe the circled alveolar space (scale bar  = 5 µm). Images are representative of 3 separate experiments.
(TIF)

Video S1 **Imaging of control lung in CD11c-eYFP^+^ animals with 655-Q-dots (red) to highlight blood vessels.** eYFP-bright dendritic cells (yellow) are readily found at, or just below the lung surface (0–50 μm deep), marked by a dense network of collagen fibrils that produce second harmonic signals (blue). DCs are also sometimes near blood vessels and actively sample the local environment (left). Deeper in the lung tissue (100–200 μm below the collagen-rich surface), alveolar macrophages that are eYFP-dim, highly spherical, non-motile and typically 10 μm or less in diameter are found associated with alveolar spaces outlined by second harmonic generation produced by collagen fibrils (right). Video durations  = 14∶10 (min:sec), 20 μm large tick marks.
(M1V)

Video S2 **A 3D rotation highlighting the loss of eYFP-bright DCs from the surface of the lung.** 24 h after influenza infection (right) compared to control lung in the same area and tissue depth (left). Collagen fibrils (blue), eYFP^+^ cells (yellow), large tick marks  = 20 μm.
(M1V)

Video S3 **Highly active eYFP^+^ DCs (yellow) at the surface of the lung, 3 days after infection.** These cells extended highly active processes and were not widely distributed but rather collected in high density nodes of eYFP^+^ DCs. Note dense collagen structure, blue. Video duration  = 32∶51 (min:sec), large tick marks  = 20 μm.
(M1V)

Video S4 **T cells (green, white tracks) and motile DCs (yellow, blue tracks) in the control lymph node (left), day 1 lymph node (center), day 3 lymph node (right).** Arrows in day 1 Video (center) point to a rare T cell in the lymph node at this time. Tracks represent 6 min of preceding T cell movement, Video duration  = 15∶49 (min:sec), and large tick marks  = 20 μm.
(M1V)

Video S5 **Three modes of T cell division imaged in the lymph node on day 3 after influenza infection were captured in 2-photon imaging.** Symmetrical contact during T cell (green) division in which both daughter cells maintain contact with the eYFP^+^ DC (yellow) supporting T cell division (left, Video duration  = 56∶14 min:sec). Polarized or asymmetrical division in which only one daughter T cell maintains contact with the DC supporting T cell division (center, Video duration  = 41∶11 min:sec), and independent division in which T cells move off of a visible APC and divide independent of eYFP^+^-DC contact (right, Video duration 43∶05 min:sec). T cells are marked by center points (red) and white tracks  = 5 min preceding T cell movement before (white) and after division (blue), large tick marks  = 10 μm.
(M1V)

Video S6 **T cells in the lung during viral clearance.** T cells (green) are first seen in the lung on day 6 (left) and are found primarily in association with eYFP^+^ DC clusters (yellow). On day 8 (right) there appear to be more T cells in the lung and these cells spend less time in close association with eYFP^+^-DCs. Tracks  = 6 min of preceding T cell movement, Video durations  = 15∶49 (min:sec), and large tick marks  = 20 μm.
(M1V)

Video S7 **T cells in lymph node during viral clearance.** In the lymph node on day 6 (left) fewer T cells are visible relative to day 3, and these remaining cells are highly motile. In the lymph node on day 8 (right) an occasional T cell division was captured by two photon imaging on day 8 (circled cell), but not on day 6. Tracks  = 6 min of preceding T cell movement, Video durations  = 25∶07 (min:sec), and large tick marks  = 20 μm.
(M1V)

Video S8 **T cells in the lung after viral clearance.** On day 10 (left) and day 14 (right) in the lung tissue T cells (green) are highly motile along collagen fibers (blue) and eYFP^+^ DCs (yellow) were evenly distributed at the surface of the lung. On Day 14 fewer T cells were visible at the surface of the lung. Tracks represent 6 min of preceding T cell movement, Video durations  = 24∶07 (min:sec), large tick marks  = 20 μm, white.
(M1V)

Video S9 **T cell and DC behavior in the deep lung after viral clearance.** On day 10 in the deep lung alveolar spaces, outlined by collagen were often sampled by motile T cells and motile eYFP^+^ DCs (yellow). In this video, (∼100 μm below the surface) the alveolar space (outlined in white) also contains a macrophage-like cell that occasionally extends a processes, Video duration  = 47∶01 (min:sec). A thick slice preparation of a whole lung lobe was used for imaging.
(M1V)

Video S10 **T cell and DC behavior in lymph node after viral clearance.** In the lymph node on day 10 (left) T cells (green) are smaller and interact less with local eYFP^+^ DCs (yellow). By day 14 (right) fewer T cells are visible relative to days 3–10 and motility had slowed significantly. Tracks represent 6 min of preceding T cell movement, Video durations  = 18∶54 (min:sec), large tick marks  = 20 μm.
(M1V)

Video S11 **AAL-R treatment: day 6 in lung.** AAL-R treatment at the time of infection led to fewer eYFP^+^ DC clusters (yellow) at the surface of the lung on day 6 (none were visible on day 3). The few visible clusters were typically less dense than those found in untreated lung tissue. T cell (green-blue) motility is slightly higher and after AAL-R treatment and T-DC contacts are shorter. Tracks represent 6 min of preceding T cell movement, Video durations  = 27∶18 (min:sec), large tick marks  = 20 μm.
(M1V)

Video S12 **AAL-R treatment: day 3 in lymph node.** Three regions of the lymph node marked by distinct distributions of eYFP^+^ DCs and distinct T cell behavior are shown. Lymphatic regions (left, region 1) where T cells (green) with low velocity and high directional persistence (green) crawl over and around areas filled with eYFP^+^ DCs (yellow) that correspond to afferent lymphatics. Clustered regions of eYFP^+^ DCs that T cells have entered and actively engage eYFP^+^ DCs, while having low directional persistence and velocity highlighting T cell confinement within the DC clusters (center, region 2). eYFP^+^ DCs deeper in the lymph node were more diffuse and T cells on day 3 in this region demonstrated the highest velocity and directional persistence of the three regions (right, region 3). Tracks represent 6 min of preceding T cell movement, Video durations  = 29∶37 (min:sec), large tick marks  = 20 μm.
(M1V)

Video S13 **AAL-R treatment: day 6 in lymph node.** In the lymph node on day 6 after infection and AAL-R treatment T cells were numerous and proliferating, similar to T cell behavior on day 3 in control imaging. Dividing T cells circled, Video duration  = 37∶10 (min:sec), large tick marks  = 20 μm.
(M1V)

## References

[1] Teijaro JR, Walsh KB, Cahalan S, Fremgen DM, Roberts E, et al (2011) Endothelial cells are central orchestrators of cytokine amplification during influenza virus infection. Cell 146: 980–991. 21925319 10.1016/j.cell.2011.08.015 PMC3176439

[2] Walsh KB, Teijaro JR, Wilker PR, Jatzek A, Fremgen DM, et al (2011) Suppression of cytokine storm with a sphingosine analog provides protection against pathogenic influenza virus. Proc Natl Acad Sci U S A 108: 12018–12023. 21715659 10.1073/pnas.1107024108 PMC3142000

[3] Walsh KB, Teijaro JR, Rosen H, Oldstone MB (2011) Quelling the storm: utilization of sphingosine-1-phosphate receptor signaling to ameliorate influenza virus-induced cytokine storm. Immunol Res 51: 15–25. 21901448 10.1007/s12026-011-8240-z

[4] La Gruta NL, Kedzierska K, Stambas J, Doherty PC (2007) A question of self-preservation: immunopathology in influenza virus infection. Immunol Cell Biol 85: 85–92. 17213831 10.1038/sj.icb.7100026

[5] Luyt CE, Combes A, Becquemin MH, Beigelman-Aubry C, Hatem S, et al.. (2012) Long-term outcomes of pandemic 2009 influenza A (H1N1)-associated severe acute respiratory distress syndrome. Chest. 10.1378/chest.11-2196 22948576

[6] Heyland DK, Groll D, Caeser M (2005) Survivors of acute respiratory distress syndrome: relationship between pulmonary dysfunction and long-term health-related quality of life. Crit Care Med 33: 1549–1556. 16003061 10.1097/01.ccm.0000168609.98847.50

[7] Marsolais D, Hahm B, Walsh KB, Edelmann KH, McGavern D, et al (2009) A critical role for the sphingosine analog AAL-R in dampening the cytokine response during influenza virus infection. Proc Natl Acad Sci U S A 106: 1560–1565. 19164548 10.1073/pnas.0812689106 PMC2635800

[8] Walsh KB, Marsolais D, Welch MJ, Rosen H, Oldstone MB (2010) Treatment with a sphingosine analog does not alter the outcome of a persistent virus infection. Virology 397: 260–269. 19962171 10.1016/j.virol.2009.08.043 PMC2821988

[9] Brun-Buisson C, Richard JC, Mercat A, Thiebaut AC, Brochard L (2011) Early corticosteroids in severe influenza A/H1N1 pneumonia and acute respiratory distress syndrome. Am J Respir Crit Care Med 183: 1200–1206. 21471082 10.1164/rccm.201101-0135OC

[10] Martin-Loeches I, Lisboa T, Rhodes A, Moreno RP, Silva E, et al (2011) Use of early corticosteroid therapy on ICU admission in patients affected by severe pandemic (H1N1)v influenza A infection. Intensive Care Med 37: 272–283. 21107529 10.1007/s00134-010-2078-z PMC7079858

[11] GeurtsvanKessel CH, Willart MA, van Rijt LS, Muskens F, Kool M, et al (2008) Clearance of influenza virus from the lung depends on migratory langerin+CD11b- but not plasmacytoid dendritic cells. J Exp Med 205: 1621–1634. 18591406 10.1084/jem.20071365 PMC2442640

[12] Cahalan MD, Parker I (2008) Choreography of cell motility and interaction dynamics imaged by two-photon microscopy in lymphoid organs. Annu Rev Immunol 26: 585–626. 18173372 10.1146/annurev.immunol.24.021605.090620 PMC2732400

[13] Germain RN, Robey EA, Cahalan MD (2012) A decade of imaging cellular motility and interaction dynamics in the immune system. Science 336: 1676–1681. 22745423 10.1126/science.1221063 PMC3405774

[14] Lindquist RL, Shakhar G, Dudziak D, Wardemann H, Eisenreich T, et al (2004) Visualizing dendritic cell networks in vivo. Nat Immunol 5: 1243–1250. 15543150 10.1038/ni1139

[15] Thornton EE, Looney MR, Bose O, Sen D, Sheppard D, et al (2012) Spatiotemporally separated antigen uptake by alveolar dendritic cells and airway presentation to T cells in the lung. J Exp Med 209: 1183–1199. 22585735 10.1084/jem.20112667 PMC3371730

[16] Ho AW, Prabhu N, Betts RJ, Ge MQ, Dai X, et al (2011) Lung CD103+ dendritic cells efficiently transport influenza virus to the lymph node and load viral antigen onto MHC class I for presentation to CD8 T cells. J Immunol 187: 6011–6021. 22043017 10.4049/jimmunol.1100987

[17] Kim TS, Braciale TJ (2009) Respiratory dendritic cell subsets differ in their capacity to support the induction of virus-specific cytotoxic CD8+ T cell responses. PLoS One 4: e4204. 19145246 10.1371/journal.pone.0004204 PMC2615220

[18] Pircher H, Burki K, Lang R, Hengartner H, Zinkernagel RM (1989) Tolerance induction in double specific T-cell receptor transgenic mice varies with antigen. Nature 342: 559–561. 2573841 10.1038/342559a0

[19] Carrat F, Vergu E, Ferguson NM, Lemaitre M, Cauchemez S, et al (2008) Time lines of infection and disease in human influenza: a review of volunteer challenge studies. Am J Epidemiol 167: 775–785. 18230677 10.1093/aje/kwm375

[20] Baccam P, Beauchemin C, Macken CA, Hayden FG, Perelson AS (2006) Kinetics of influenza A virus infection in humans. J Virol 80: 7590–7599. 16840338 10.1128/JVI.01623-05 PMC1563736

[21] Gonzalez SF, Lukacs-Kornek V, Kuligowski MP, Pitcher LA, Degn SE, et al (2010) Capture of influenza by medullary dendritic cells via SIGN-R1 is essential for humoral immunity in draining lymph nodes. Nat Immunol 11: 427–434. 20305659 10.1038/ni.1856 PMC3424101

[22] Vinay DS, Kwon BS (2010) CD11c+CD8+ T cells: two-faced adaptive immune regulators. Cell Immunol 264: 18–22. 20620256 10.1016/j.cellimm.2010.05.010

[23] de Heusch M, Blocklet D, Egrise D, Hauquier B, Vermeersch M, et al (2007) Bidirectional MHC molecule exchange between migratory and resident dendritic cells. J Leukoc Biol 82: 861–868. 17634280 10.1189/jlb.0307167

[24] Allan RS, Waithman J, Bedoui S, Jones CM, Villadangos JA, et al (2006) Migratory dendritic cells transfer antigen to a lymph node-resident dendritic cell population for efficient CTL priming. Immunity 25: 153–162. 16860764 10.1016/j.immuni.2006.04.017

[25] Belz GT, Smith CM, Kleinert L, Reading P, Brooks A, et al (2004) Distinct migrating and nonmigrating dendritic cell populations are involved in MHC class I-restricted antigen presentation after lung infection with virus. Proc Natl Acad Sci U S A 101: 8670–8675. 15163797 10.1073/pnas.0402644101 PMC423253

[26] Moltedo B, Li W, Yount JS, Moran TM (2011) Unique type I interferon responses determine the functional fate of migratory lung dendritic cells during influenza virus infection. PLoS Pathog 7: e1002345. 22072965 10.1371/journal.ppat.1002345 PMC3207893

[27] Lawrence CW, Braciale TJ (2004) Activation, differentiation, and migration of naive virus-specific CD8+ T cells during pulmonary influenza virus infection. J Immunol 173: 1209–1218. 15240712 10.4049/jimmunol.173.2.1209

[28] Lanzavecchia A, Sallusto F (2004) Lead and follow: the dance of the dendritic cell and T cell. Nat Immunol 5: 1201–1202. 15549117 10.1038/ni1204-1201

[29] Matheu MP, Su Y, Greenberg ML, Blanc CA, Parker I, et al (2012) Toll-like receptor 4-activated B cells out-compete Toll-like receptor 9-activated B cells to establish peripheral immunological tolerance. Proc Natl Acad Sci U S A 109: E1258–1266. 22511718 10.1073/pnas.1205150109 PMC3356607

[30] Mempel TR, Henrickson SE, Von Andrian UH (2004) T-cell priming by dendritic cells in lymph nodes occurs in three distinct phases. Nature 427: 154–159. 14712275 10.1038/nature02238

[31] Miller MJ, Safrina O, Parker I, Cahalan MD (2004) Imaging the single cell dynamics of CD4+ T cell activation by dendritic cells in lymph nodes. J Exp Med 200: 847–856. 15466619 10.1084/jem.20041236 PMC2213293

[32] Castellino F, Huang AY, Altan-Bonnet G, Stoll S, Scheinecker C, et al (2006) Chemokines enhance immunity by guiding naive CD8+ T cells to sites of CD4+ T cell-dendritic cell interaction. Nature 440: 890–895. 16612374 10.1038/nature04651

[33] McGill J, Legge KL (2009) Cutting edge: contribution of lung-resident T cell proliferation to the overall magnitude of the antigen-specific CD8 T cell response in the lungs following murine influenza virus infection. J Immunol 183: 4177–4181. 19767567 10.4049/jimmunol.0901109 PMC2762786

[34] Lawrence CW, Ream RM, Braciale TJ (2005) Frequency, specificity, and sites of expansion of CD8+ T cells during primary pulmonary influenza virus infection. J Immunol 174: 5332–5340. 15843530 10.4049/jimmunol.174.9.5332

[35] Ballesteros-Tato A, Leon B, Lund FE, Randall TD (2010) Temporal changes in dendritic cell subsets, cross-priming and costimulation via CD70 control CD8(+) T cell responses to influenza. Nat Immunol 11: 216–224. 20098442 10.1038/ni.1838 PMC2822886

[36] Ho AW, Prabhu N, Betts RJ, Ge MQ, Dai X, et al (2011) Lung CD103+ dendritic cells efficiently transport influenza virus to the lymph node and load viral antigen onto MHC class I for presentation to CD8 T cells. J Immunol 187: 6011–6021. 22043017 10.4049/jimmunol.1100987

[37] Lambrecht BN, Hammad H (2012) Lung dendritic cells in respiratory viral infection and asthma: from protection to immunopathology. Annu Rev Immunol 30: 243–270. 22224777 10.1146/annurev-immunol-020711-075021

[38] del Rio ML, Rodriguez-Barbosa JI, Kremmer E, Forster R (2007) CD103- and CD103+ bronchial lymph node dendritic cells are specialized in presenting and cross-presenting innocuous antigen to CD4+ and CD8+ T cells. J Immunol 178: 6861–6866. 17513734 10.4049/jimmunol.178.11.6861

[39] Kumagai Y, Takeuchi O, Kato H, Kumar H, Matsui K, et al (2007) Alveolar macrophages are the primary interferon-alpha producer in pulmonary infection with RNA viruses. Immunity 27: 240–252. 17723216 10.1016/j.immuni.2007.07.013

[40] Braciale TJ, Sun J, Kim TS (2012) Regulating the adaptive immune response to respiratory virus infection. Nat Rev Immunol 12: 295–305. 22402670 10.1038/nri3166 PMC3364025

[41] del Rio ML, Bernhardt G, Rodriguez-Barbosa JI, Forster R (2010) Development and functional specialization of CD103+ dendritic cells. Immunol Rev 234: 268–281. 20193025 10.1111/j.0105-2896.2009.00874.x

[42] Chang JT, Palanivel VR, Kinjyo I, Schambach F, Intlekofer AM, et al (2007) Asymmetric T lymphocyte division in the initiation of adaptive immune responses. Science 315: 1687–1691. 17332376 10.1126/science.1139393

[43] Ciocca ML, Barnett BE, Burkhardt JK, Chang JT, Reiner SL (2012) Cutting edge: Asymmetric memory T cell division in response to rechallenge. J Immunol 188: 4145–4148. 22467651 10.4049/jimmunol.1200176 PMC3331961

[44] Beuneu H, Lemaitre F, Deguine J, Moreau HD, Bouvier I, et al (2010) Visualizing the functional diversification of CD8+ T cell responses in lymph nodes. Immunity 33: 412–423. 20850354 10.1016/j.immuni.2010.08.016

[45] Kang SS, Herz J, Kim JV, Nayak D, Stewart-Hutchinson P, et al (2011) Migration of cytotoxic lymphocytes in cell cycle permits local MHC I-dependent control of division at sites of viral infection. J Exp Med 208: 747–759. 21464219 10.1084/jem.20101295 PMC3135345

[46] Matheu MP, Beeton C, Garcia A, Chi V, Rangaraju S, et al (2008) Imaging of effector memory T cells during a delayed-type hypersensitivity reaction and suppression by Kv1.3 channel block. Immunity 29: 602–614. 18835197 10.1016/j.immuni.2008.07.015 PMC2732399

[47] Kim TS, Hufford MM, Sun J, Fu YX, Braciale TJ (2010) Antigen persistence and the control of local T cell memory by migrant respiratory dendritic cells after acute virus infection. J Exp Med 207: 1161–1172. 20513748 10.1084/jem.20092017 PMC2882836

[48] Zammit DJ, Turner DL, Klonowski KD, Lefrancois L, Cauley LS (2006) Residual antigen presentation after influenza virus infection affects CD8 T cell activation and migration. Immunity 24: 439–449. 16618602 10.1016/j.immuni.2006.01.015 PMC2861289

[49] Halle S, Dujardin HC, Bakocevic N, Fleige H, Danzer H, et al (2009) Induced bronchus-associated lymphoid tissue serves as a general priming site for T cells and is maintained by dendritic cells. J Exp Med 206: 2593–2601. 19917776 10.1084/jem.20091472 PMC2806625

[50] Jelley-Gibbs DM, Brown DM, Dibble JP, Haynes L, Eaton SM, et al (2005) Unexpected prolonged presentation of influenza antigens promotes CD4 T cell memory generation. J Exp Med 202: 697–706. 16147980 10.1084/jem.20050227 PMC2212871

[51] Seder RA, Ahmed R (2003) Similarities and differences in CD4+ and CD8+ effector and memory T cell generation. Nat Immunol 4: 835–842. 12942084 10.1038/ni969

[52] Masopust D, Ahmed R (2004) Reflections on CD8 T-cell activation and memory. Immunol Res 29: 151–160. 15181278 10.1385/IR:29:1-3:151

[53] Marsolais D, Hahm B, Edelmann KH, Walsh KB, Guerrero M, et al (2008) Local not systemic modulation of dendritic cell S1P receptors in lung blunts virus-specific immune responses to influenza. Mol Pharmacol 74: 896–903. 18577684 10.1124/mol.108.048769 PMC2574812

[54] McGavern DB, Christen U, Oldstone MB (2002) Molecular anatomy of antigen-specific CD8(+) T cell engagement and synapse formation in vivo. Nat Immunol 3: 918–925. 12352968 10.1038/ni843 PMC2481514

[55] Bergner A, Sanderson MJ (2002) Acetylcholine-induced calcium signaling and contraction of airway smooth muscle cells in lung slices. J Gen Physiol 119: 187–198. 11815668 10.1085/jgp.119.2.187 PMC2233801

[56] Looney MR, Thornton EE, Sen D, Lamm WJ, Glenny RW, et al (2010) Stabilized imaging of immune surveillance in the mouse lung. Nat Methods 8: 91–96. 21151136 10.1038/nmeth.1543 PMC3076005

[57] Kreisel D, Nava RG, Li W, Zinselmeyer BH, Wang B, et al (2010) In vivo two-photon imaging reveals monocyte-dependent neutrophil extravasation during pulmonary inflammation. Proc Natl Acad Sci U S A 107: 18073–18078. 20923880 10.1073/pnas.1008737107 PMC2964224

[58] Cohen J (1988) Statistical power analysis for the behavioral sciences. Hillsdale, N.J.: L. Erlbaum Associates.

