# Supplementary figures and images for: Three Phases of CD8 T Cell Response in the Lung Following H1N1 Influenza Infection and Sphingosine 1 Phosphate Agonist Therapy
Source: PLoS One. 2013 Mar 22;8(3):e58033. doi: 10.1371/journal.pone.0058033 (PMC3606384; doi:10.1371/journal.pone.0058033)

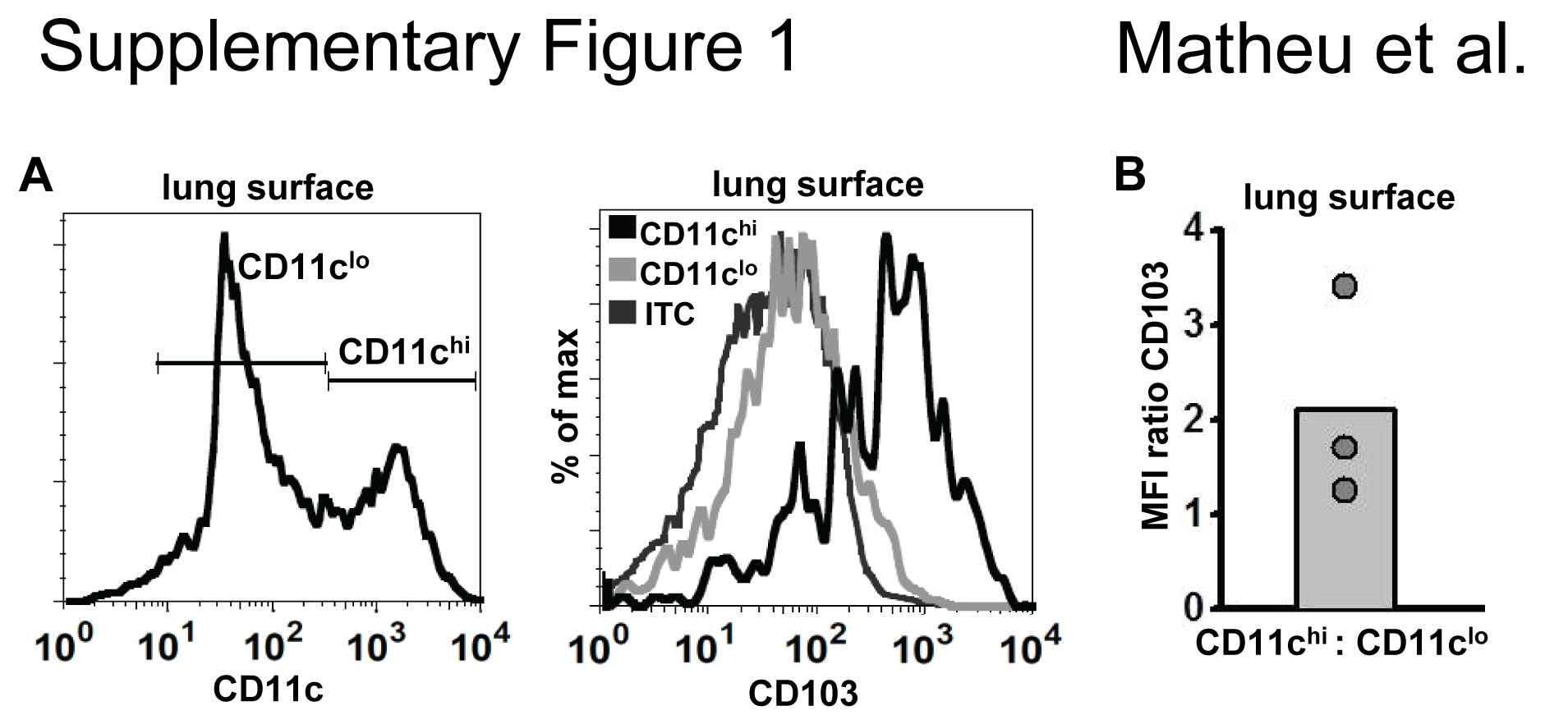

Supplement: Figure S1 — CD11chi DCs near the lung surface are CD103+. (A) FACS analysis of the lung surface gated on a high forward scatter profile (left), revealing CD11chi and CD11clo populations. CD103 expression in these two populations (right). (B) Expression of CD103 in CD11chi, relative to CD11clo cells on the surface of the lung (ratio = 2.1±0.7). Data are representative of 3 separate experiments. (TIF) [file pone.0058033.s001.tif]

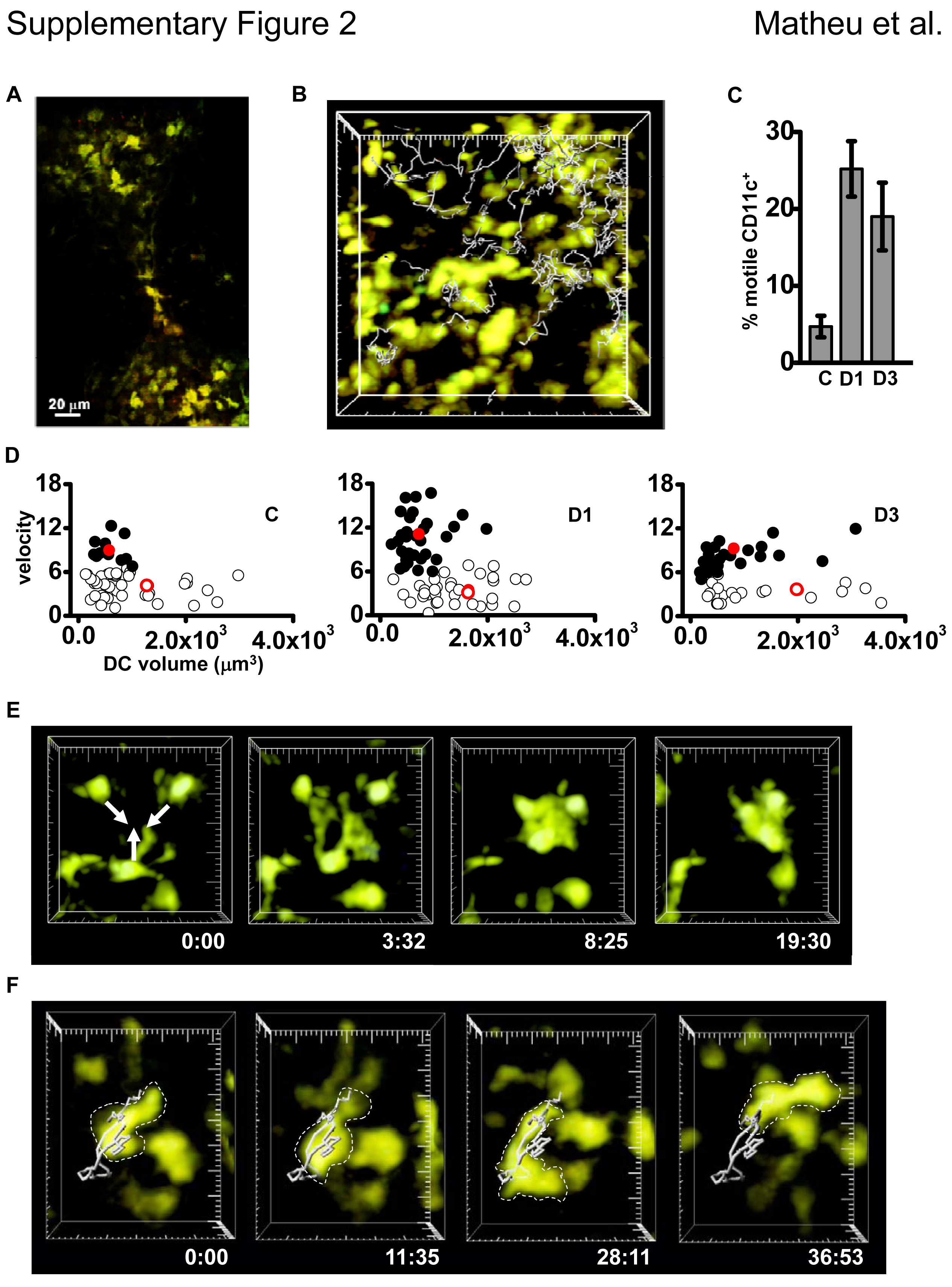

Supplement: Figure S2 — Early DC infiltration and behavior in the lymph node. (A) Compiled image of DCs in the lymph node on day 1 show eYFP+ cells outlining afferent lymphatic space. (B) Tracks of motile DCs deep in the lymph node on day 1 post-infection (46∶43 min:sec), large tick marks = 10 μm. (C) Percent motile DCs (eYFP+) cells in the lymph node is significantly higher on day 1 (25.2±3.6%, p<0.01) and day 3 (19.0±4.2%) compared to the percent of motile DCs in the control lymph node (4.7±1.4%, p<0.01, both). (D) Dendritic cell volume v. track velocity plots show that cells with an average velocity >6 μm/min have significantly lower volume (closed circles) relative to eYFP+ DCs moving <6 μm/min (open circles). In control lymph nodes (volume motile = 570±75 μm3, sessile = 1209±287 μm3, p<0.01), day 1 (volume motile = 715±68 μm3, sessile = 1643±206 μm3, p<0.01) and day 3 (volume motile = 803±140 μm3, sessile = 2002±328 μm3, p<0.01). Mean values for each group are denoted by the red dot. (E) DCs exhibit several different behaviors on days 1 and 3 in the lymph node, here on day 3 DCs move together to form a sessile cluster. (F) A motile DC engages, and crawls on and around a sessile cluster of DCs, large tick marks = 5 μm; track duration = 36∶53 min:sec. Data were compiled from 4–6 separate experiments; each dot represents measurements taken from a single cell. (TIF) [file pone.0058033.s002.tif]

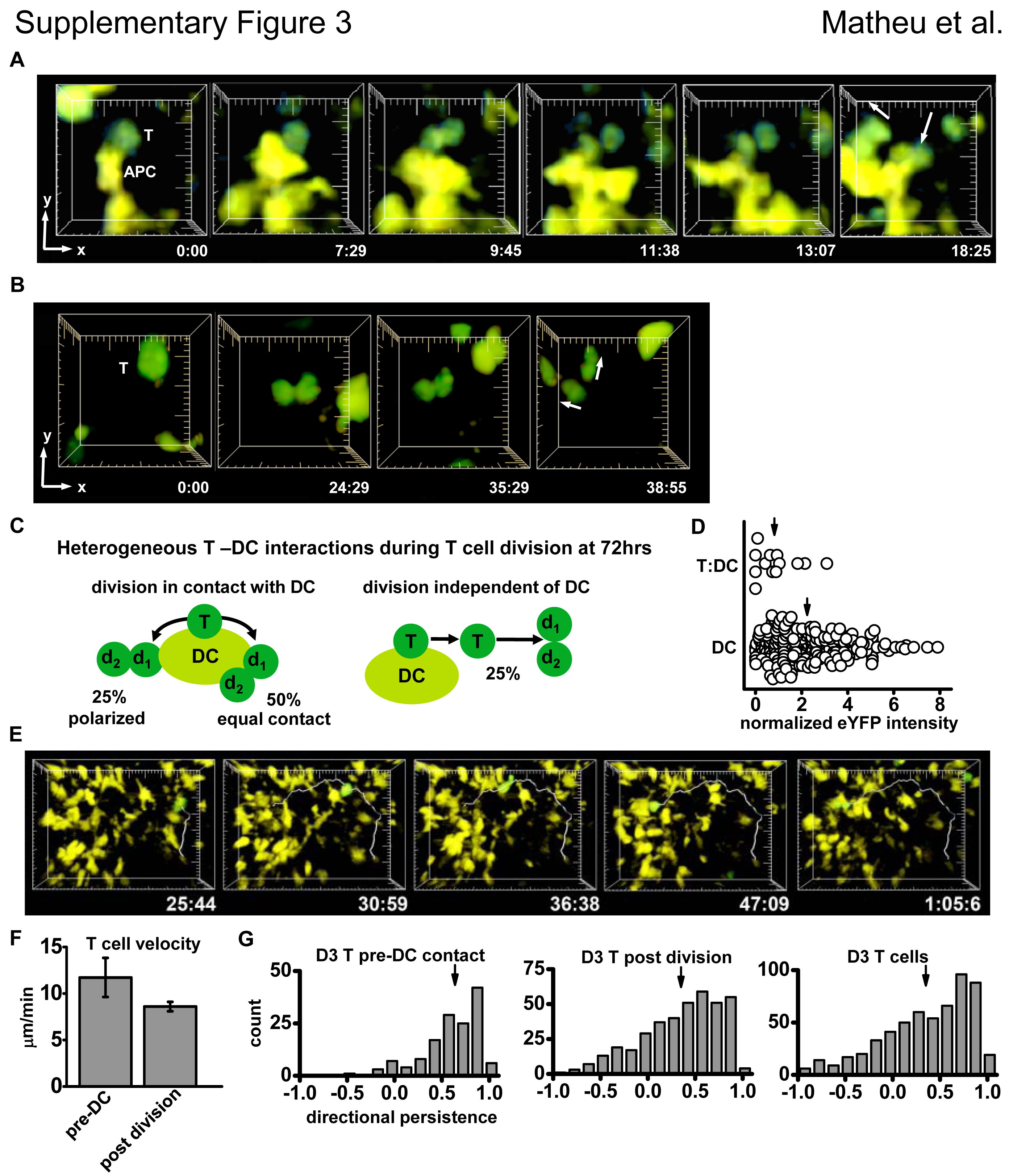

Supplement: Figure S3 — Characteristics of dividing T cells in the lymph node on day 3. (A) Images of a CD8+ T cell dividing in a polarized manner while in contact with a sessile DC. White arrows in the last frame point to the direction of movement taken by the daughter cells. (B) Images of a CD8+ T cell division while not in contact with a sessile DC. (C) Analysis of 20 examples of cell division in 3 separate lymph nodes, 3 days after influenza infection. Most cells divide while in contact with a sessile DC. (D) Brightness of DCs in contact with T cells leading to division, and alone (mean relative brightness = 0.9±0.26) normalized to all DCs in the imaging volume (mean relative brightness ratio = 2.4±0.1), where the dimmest visible cell = 0; n = 3 separate experiments. (E) Time-lapse images of a CD8+ T cell on day 3. The cell makes a sharp turn and moves in a highly directional manner prior to division on a sessile DC; track duration = 49∶32 min:sec. (F) T cell velocity prior to contacting DC and dividing (11.4±1.8 μm/min, n = 8 tracks) and daughter cell velocity after detachment from the DC (8.4±0.5 μm/min, n = 16 tracks, p = 0.04). (G) Analysis of T cell directional persistence (5–10 min) prior to contact with a DC on which division occurs. Counts represent the directional persistence of every two steps taken by the T cell. T cells showed high directional persistence (0.63±0.05, n = 8 cells), compared to both daughter T cell motility (n = 16 cells) after division (0.35±0.04, p<0.01), and pooled day 3 T cells (0.36±0.02, p<0.01, n = 4 separate experiments for all division data). (TIF) [file pone.0058033.s003.tif]

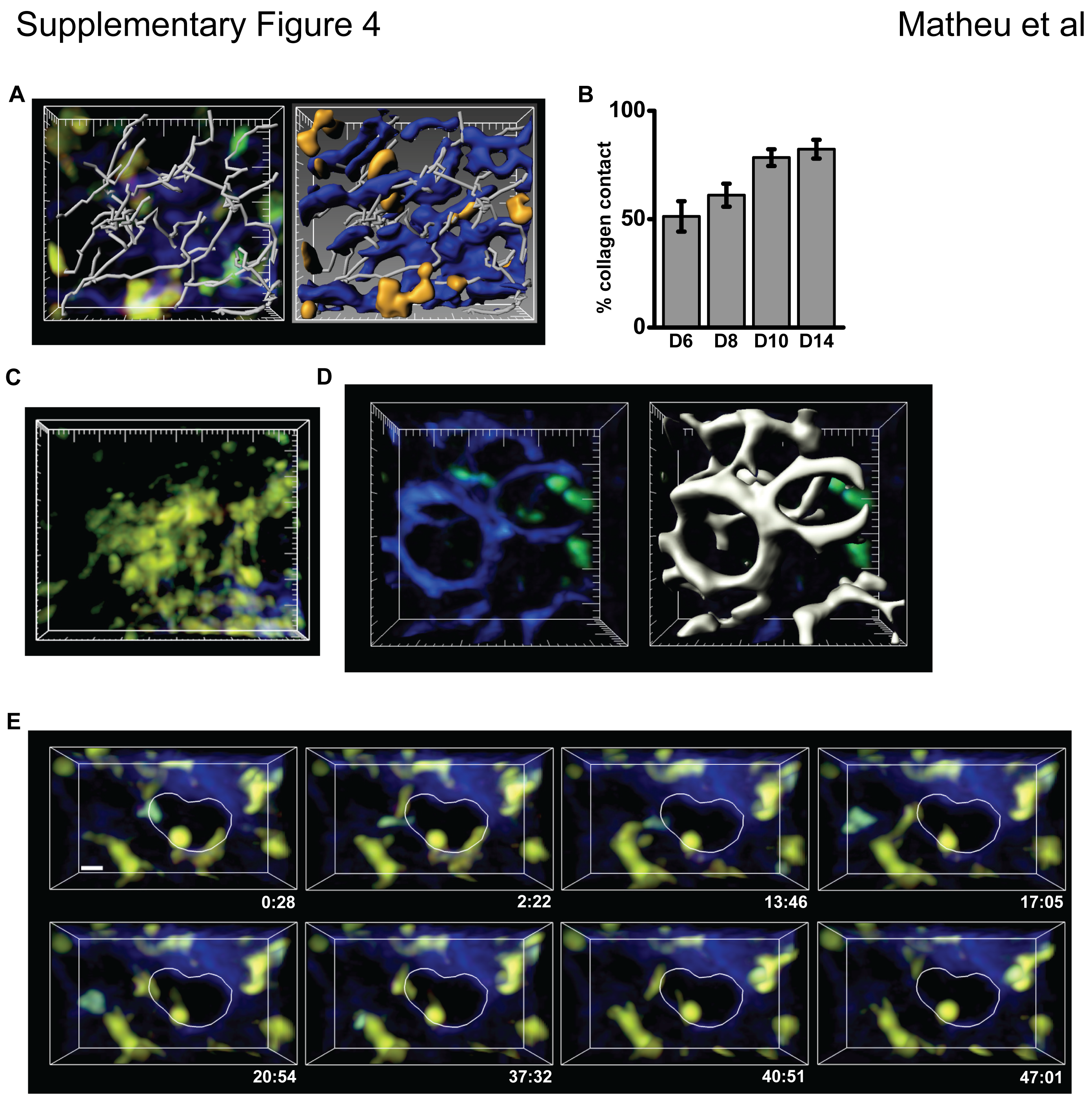

Supplement: Figure S4 — T cell motility and behavior in deep lung parenchyma. (A) T cell tracks (grey) in the lung on day 10 over 40 minutes of imaging demonstrate that T cells preferentially crawl along collagen fibrils embedded with eYFP+ DCs (left), represented in a space filling model where collagen fibrils are blue, APCs are gold and T cell tracks are grey (right; large ticks = 10 µm). (B) Percent of time T cells in the lung spend in contact with a visible collagen fiber steadily increases between day 6 (51±7%) and day 14 (82±4%, p<0.01). (C) Cluster of DCs on day 10 in the lung were found deeper in the lung tissue (100 microns from the surface), than clusters of DCs imaged at earlier time points. (D) Close up of collagen bands (blue) supporting alveolar sacs in the deep lung (and outlining alveolar space) and T cells (green) that occasionally enter the alveolar space. (E) A series of images where in both a T cell (green, panels 0∶28 to 13∶46 min), a motile DC (yellow, panels 17∶05 to 37∶32 min), and alveolar macrophage (yellow, panels 20∶54 to 40∶51 min) probe the circled alveolar space (scale bar = 5 µm). Images are representative of 3 separate experiments. (TIF) [file pone.0058033.s004.tif]
